# MDM2 provides TOP2 poison resistance by promoting proteolysis of TOP2βcc in a p53-independent manner

**DOI:** 10.1038/s41419-024-06474-3

**Published:** 2024-01-23

**Authors:** Jianfeng Shu, Jinni Jiang, Xiaofang Wang, Xuejie Yang, Guofang Zhao, Ting Cai

**Affiliations:** 1 Department of Thoracic Surgery, Ningbo No.2 Hospital, Ningbo, 315010 Zhejiang China; 2 Ningbo Institute of Life and Health Industry, University of Chinese Academy of Sciences, Ningbo, 315000 Zhejiang China

**Keywords:** Ubiquitin ligases, Chemotherapy, Proteases

## Abstract

DNA topoisomerase II (TOP2) is an enzyme that performs a critical function in manipulating DNA topology during replication, transcription, and chromosomal compaction by forming a vital intermediate known as the TOP2-DNA cleavage complex (TOP2cc). Although the TOP2cc is often transient, stabilization can be achieved by TOP2 poisons, a family of anti-cancer chemotherapeutic agents targeting TOP2, such as etoposide (VP-16), and then induce double-strand breaks (DSBs) in cellular DNA. TOP2cc first needs to be proteolyzed before it can be processed by TDP2 for the removal of these protein adducts and to produce clean DNA ends necessary for proper repair. However, the mechanism by which TOP2βcc is proteolyzed has not been thoroughly studied. In this study, we report that after exposure to VP-16, MDM2, a RING-type E3 ubiquitin ligase, attaches to TOP2β and initiates polyubiquitination and proteasomal degradation. Mechanistically, during exposure to VP-16, TOP2β binds to DNA to form TOP2βcc, which promotes MDM2 binding and subsequent TOP2β ubiquitination and degradation, and results in a decrease in TOP2βcc levels. Biologically, MDM2 inactivation abrogates TOP2β degradation, stabilizes TOP2βcc, and subsequently increases the number of TOP2β-concealed DSBs, resulting in the rapid death of cancer cells via the apoptotic process. Furthermore, we demonstrate the combination activity of VP-16 and RG7112, an MDM2 inhibitor, in the xenograft tumor model and in situ lung cancer mouse model. Taken together, the results of our research reveal an underlying mechanism by which MDM2 promotes cancer cell survival in the presence of TOP2 poisons by activating proteolysis of TOP2βcc in a p53-independent manner, and provides a rationale for the combination of MDM2 inhibitors with TOP2 poisons for cancer therapy.

## Introduction

Eukaryotic DNA is coiled around histone octamers to form chromatin, which is described as a “string of beads” [1]. To decrease strand helix stress, adjustments to DNA’s conformation and topological structure are required for various bioactivities, including DNA transcription, replication, and chromosome compression [2]. To alleviate this helical stress, homodimeric enzymes called DNA topoisomerases II (TOP2) nick both DNA strands, resulting in TOP2-concealed double-strand breaks (DSBs), and then transfer an intact double strand through the transitory break. In this step, a crucial intermediate called the TOP2-DNA cleavage complex (TOP2cc) is formed whenever the TOP2 homodimer active tyrosine forms a covalent bond with the 5’-end of each double-strand DNA [3]. The TOP2cc structure typically has a short duration and can be reversed. TOP2 re-ligates the ends in situ once another molecule of DNA has gone through the break spot without the aid of any other enzymes.

DNA topoisomerase II (TOP2) is found in humans in two isoforms, TOP2α and TOP2β. TOP2α plays a primary role in mediating cellular proliferation, while TOP2β is predominantly involved in the process of cell transcription and differentiation [4]. Beyond their essential physiological activities, TOP2α and TOP2β are treatment targets for widely prescribed anti-cancer medications, particularly, TOP2 poisons, including etoposide, doxorubicin, and teniposide [4, 5]. These anti-cancer medicines work by inhibiting TOP2’s DNA religation function, which stabilizes the TOP2-DNA covalent complex and ultimately leads to the destruction of TOP2cc by 26 S proteasome. As a result, the TOP2-masked DSBs are exposed, transformed into true DSBs, and trigger a DNA damage response (DDR) [6]. Due to this mechanism, patients treated with TOP2 poisons have a high incidence of secondary malignancies and cardiotoxicity [6, 7]. Additionally, TOP2 degradation may cause a drop in cytotoxic TOP2cc levels in cancerous cells, which in turn can contribute to the development of resistance to anti-cancer medicines [8]. Previous research illustrates that TOP2α is required for the cytotoxicity of TOP2 poisons, while DSBs and rearrangements in DNA sequence may be traced back to the degradation of TOP2βcc [9]. Moreover, TOP2β is preferentially degraded over TOP2α. Recently, our group reported that SCF^β-TrCP^ ubiquitin ligase partly mediated TOP2β degradation induced by teniposide, as evidenced by β-TrCP knockout partially suppressing the polyubiquitination of TOP2β and extending its protein half-life [10]. Second, these results imply that there may be other unidentified pathways that control TOP2β stability. It is worth noting that overexpressing MDM2 in cancer cells reduces DNA DSBs caused by VP-16 therapy, and MDM2-amplified cancer cells show selective resistance to TOP2 poisons and not to other genotoxic agents [11]. Moreover, DNA damage induced by TOP2 poisons may be selectively mitigated depending on the levels of MDM2 and an intact ubiquitin ligase activity, but not on levels of p53 or TOP2α [11]. Consistently, it was found that when miR-181b binds to the 3’-UTR of MDM2, its expression is downregulated, making glioma cells more sensitive to teniposide [12]. However, the underlying molecular mechanisms remain elusive. We therefore hypothesized that MDM2 could perform the E3 ligase function to regulate TOP2βcc levels when treated with a TOP2 poison.

The MDM2 protein is a well-studied oncogene product with a crucial function in cancer cell survival, invasiveness, and therapeutic resistance [13]. As the most often amplified gene in a wide range of human malignancies, MDM2’s significance in tumor growth cannot be underestimated [14]. Extensive investigations have shown that MDM2 primarily regulates the p53 pathway negatively by forming a complex with p53, repressing p53-induced transcription, and triggering ubiquitination and subsequent destruction of p53 via its E3 ligase function mediated by RING finger [15]. In addition, MDM2 can also mediate the shuttling of p53 out of the nucleus and p53 conjugation with NEDD8 and SUMO-2/3 to regulate its transcriptional activity [16, 17]. In addition to p53, several other interaction molecules and/or ubiquitination substrates for MDM2 have been identified that also appear to affect carcinogenesis. For instance, cancer cells’ translation of VEGF, XIAP, MYCN, and Slug mRNAs can be regulated by MDM2’s C-terminal RING domain [15]. MDM2 acts as an E3 ubiquitin ligase, mediating the ubiquitination and destruction of FOXO3a and HDAC1 [18, 19]. Furthermore, MDM2 was found to promote the destruction of the tumor suppressors Rb and p21 via a proteasome-dependent ubiquitin-independent pathway [20, 21]. Given its importance in promoting tumorigenesis, MDM2 has become a highly sought-after tumor therapeutic target. RG7112 and AMG 232 are examples of small-molecule MDM2 inhibitors [22], which block the interaction between MDM2 and p53 and inhibit MDM2 from acting as a ubiquitin ligase, becoming the subject of preclinical and clinical research on their potential use in cancer therapy [22]. Therefore, identifying additional molecules that interact with MDM2 would be crucial for further understanding of its oncogenic activity and may result in the identification of new targets for cancer treatment.

Furthermore, it is well established that the mammalian p53 tumor suppressor functions as a guardian of the genome by detecting DNA DSBs caused by different types of cellular stress and regulating the DNA repair process. MDM2 negatively regulates p53, which suggests that it has p53-dependent functions and thus contributes to genomic instability [23]. In addition, MDM2 was also found to regulate the DNA DSBs repair response and genome stability without involving p53 via ubiquitination of HBP1 (a transcription factor) and interacting with Nbs1, a component of the Mre11/Rad50/Nbs1 DNA DSBs repair complex [24, 25]. However, it is still unclear whether the MDM2 ubiquitin ligase controls the DDR and DNA repair induced by TOP2 poisons and, if so, the underlying mechanisms.

Herein, we report that MDM2 performs the function of an E3 ubiquitin ligase for TOP2β. Upon VP-16 treatment, MDM2 downregulates TOP2β protein levels via the ubiquitin-mediated proteasome pathway. Both MDM2 and TOP2β colocalize in the nucleus and interact with each other in vivo and in vitro. Additionally, the intact ubiquitin ligase function of MDM2 is required for downregulating TOP2β. By employing siRNA-mediated knockdown of p53 in A549 cells, we further confirmed that the levels of p53 do not impact the interaction between MDM2 and TOP2β, nor do they affect the degradation of the TOP2β. RG7112, an MDM2 inhibitor, prevents MDM2 from interacting with TOP2β, thereby blocking TOP2β from being ubiquitinated. Furthermore, we demonstrate that MDM2-mediated TOP2β degradation is not due to DSBs, as MDM2 also promotes TOP2β degradation induced by a TOP2 catalytic inhibitor ICRF-193, which inhibits topoisomerase reactions at other steps of the TOP2 reaction cycle without the formation of a DNA lesion [26]. Additionally, MDM2 inactivation via genetic depletion and pharmacological methods not only elevates TOP2βcc levels but also inhibits the DNA damage response generated by VP-16 treatment. Consequently, inhibition of TOP2β degradation by an MDM2 inhibitor causes cancer cells to be more susceptible to VP-16 by promoting cancer cell apoptosis. Finally, we further investigated the efficacy and safety of combining RG7112 and VP-16 chemotherapy using an in vivo xenograft tumor model and in situ mouse model for lung cancer to explore the therapeutic outcome of the combinatorial regimen for lung cancer. Taken together, our results suggest a novel strategy to enhance the effectiveness of TOP2-targeted anti-cancer therapies and reduce side effects by combining them with MDM2 inhibitors to prevent TOP2βcc proteolysis.

## Results

### MDM2 controls the abundance of TOP2β but not TOP2α

As is well known, the p53 tumor suppressor protects the stability of the human genome when cells are experiencing DNA DSBs caused by a variety of cell stress types, whereas MDM2 is known to negatively regulate p53 and has been shown to ubiquitylate p53 and suppress its transcriptional activity. Remarkably, the MDM2 gene is transcriptionally activated by p53 [27]. Therefore, to rule out the possible involvement of p53 in regulating TOP2β abundance, we chose to study the H1299 cell line, which has a homozygous partial deletion of the p53 gene, and the A549 cell line, which possesses a wild-type copy of p53.

**Fig. 1:**
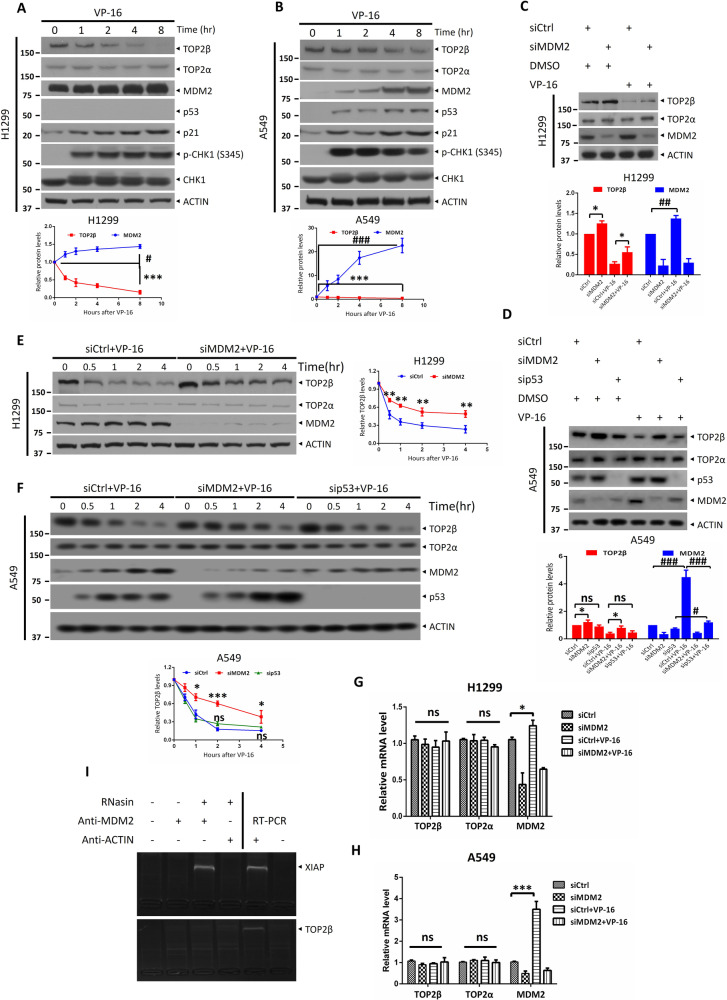
MDM2 controls the abundance of TOP2β but not TOP2α. **A**, **B** TOP2 poison VP-16 triggers TOP2β degradation but does not affect TOP2α. H1299 (**A**) and A549 (**B**) cells were treated with VP-16 (200 μM) for indicated time periods, collected, and subjected to immunoblotting (IB) with the indicated antibodies (Abs). **C**, **D** MDM2 but not p53 knockdown counteracted the VP-16-induced reduction in TOP2β. siRNA directed against MDM2, p53 or control scrambled siRNA were transfected into H1299 (**C**) or A549 (**D**), then left untreated or subjected to VP-16 (200 μM) treatment for 2 h, and finally extracted for IB using the appropriate Abs. **E**, **F** MDM2 but not p53 silencing inhibits the TOP2β degradation induced by VP-16 administration in a time-dependent manner. Cells transfected with siRNA oligos targeting MDM2, p53 or scrambled control siRNA were treated with VP-16 for the selected time periods, and then IB was performed with the indicated Abs. **G**, **H** The levels of TOP2β and TOP2α mRNA were unaffected by MDM2 knockdown. Cells treated as in (**C**, **D**) were harvested, and RNA was isolated and processed via qRT-PCR. The results are the average of 3 different replications; error bars represent SEM. **I** MDM2 can bind to XIAP mRNA but not TOP2β mRNA. RNA-binding buffer was used to prepare A549 cell extracts when an RNase inhibitor (RNasin) was present. After coimmunoprecipitation with anti-MDM2 and anti-actin (negative control), the XIAP and TOP2β mRNA was measured via qRT‒PCR analysis and nucleic acid electrophoresis using XIAP-specific primers (forward, 5′-ATG ACT TTT AAC AG TTT TGA AGG-3′; reverse, 5′-GCT CGT GCC AGT GTT GAT GCT G-3′) and TOP2β-specific primers (forward, 5′- ATG GCC AAG TCG GGT GGC TGC GG-3′; reverse, 5′-TCT TCT GAT ACA CTC TCT CAA CA-3′). All experiments were independently repeated three times. Densitometry quantification was performed with Image J, and the quantification results are shown. *p < 0.05; **p < 0.01; ***p < 0.001; ^#^*p* < 0.05; ^##^*p* < 0.01; ^###^*p* < 0.001; ns no significant.

In agreement with prior research, we discovered that treatment with the TOP2 poison VP-16 significantly stimulated the TOP2β degradation in A549 and H1299 cells but had a negligible impact on the levels of TOP2α (Fig. 1A, B). Additionally, it was reported that DNA damage signals triggered the rapid degradation of the MDM2 protein, allowing p53 accumulation and full activation [28]. However, surprisingly, upon VP-16 stimulation, the degree of expression of MDM2 was substantially increased in the experimental cell lines, especially in the A549 cells, along with increased protein levels of p53 (Fig. 1B). This may be explained by the fact that the levels of MDM2 protein are maintained in homeostasis by a constant cycle of synthesis and degradation. Following DNA damage, a dual mechanism unfolds. Firstly, it instigates the degradation of the MDM2 protein. Simultaneously, this triggers the activation of wild-type p53, thereby orchestrating the transcription of the MDM2 gene. Consequently, this intricate interplay leads to the early induction of MDM2 as evidenced by the enhanced mRNA levels of MDM2 (Fig. 1H). In addition, a slight upregulation of MDM2 mRNA and protein levels was also observed in p53-null H1299 cells (Fig. 1A, G), indicating that other unidentified transcription factors are involved in regulating MDM2 upon VP-16-induced DNA damage. Then, we checked to determine whether MDM2 had a detrimental effect on TOP2β levels. In fact, we discovered that the knockdown of MDM2 using siRNA oligos greatly attenuated the downregulation of TOP2β induced by VP-16 stimulation. (Fig. 1C, D) and caused a time-dependent elevation in TOP2β protein levels (Fig. 1E, F). In addition, to further exclude the possibility of p53 involvement in regulating the abundance of TOP2β protein, we simultaneously examined whether siRNA-mediated knockdown of p53 in A549 cells would affect the degradation of TOP2β protein. The results revealed that the knockdown of p53 did not attenuate the VP-16-induced downregulation of TOP2β, nor did it induce a time-dependent elevation in TOP2β protein levels (Fig. 1D, F). Furthermore, we evaluated if MDM2 alters the levels of TOP2β protein by regulating TOP2β mRNA levels. Figure 1G, H illustrate that the TOP2β and TOP2α mRNA levels did not change in response to MDM2 knockdown and VP-16 treatment. As mentioned above, the C-terminal RING domain of MDM2 can also bind to the mRNAs of substrate proteins to regulate their translation. Indeed, our RNA-binding protein immunoprecipitation assay showed that MDM2 directly interacts with XIAP mRNA, as a positive control, but not TOP2β mRNA (Fig. 1I), indicating that MDM2 does not negatively regulate TOP2β at the translational level. In summary, the above findings illustrated that MDM2 regulates TOP2β abundance through a posttranscriptional mechanism.

### MDM2 interacts with TOP2β to regulate its ubiquitination and degradation in response to VP-16 stimuli

**Fig. 2:**
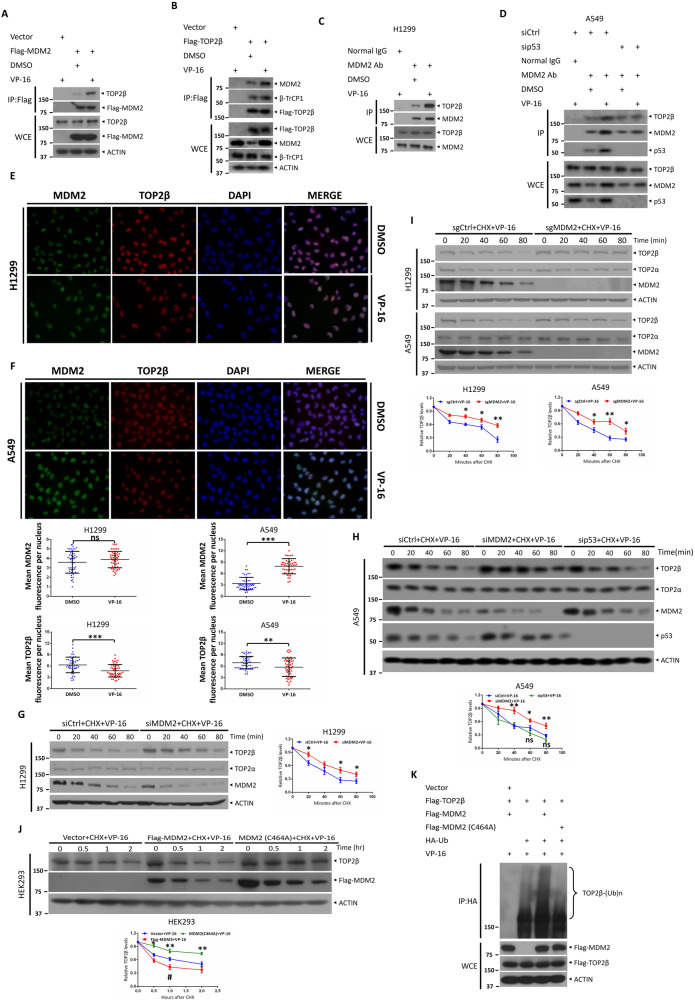
MDM2 interacts with TOP2β to regulate its ubiquitination and degradation in response to VP-16 stimuli. **A** Ectopically expressed MDM2 binds to endogenous TOP2β. **B** Ectopically expressed TOP2β binds to endogenous MDM2. After transfection with the indicated plasmids for 48 h, HEK293 cells were either left untreated or treated with VP-16 and MG132 (20 μM) for 5 h, followed by IP with FLAG beads and IB with the indicated Abs. **C** MDM2 binds to endogenous TOP2β, and TOP2β binds to endogenous MDM2. H1299 cells were either left untreated or treated with VP-16 and MG132 (20 μM) for 5 h, cells were then lysed and subjected to IP with MDM2 antibody or normal rabbit immunoglobulin (IgG), and then, IB was performed with the indicated Abs. **D** Knockdown of p53 did not exert any discernible impact on the interaction between MDM2 and TOP2β. A549 cells transfected with siRNA oligos targeting MDM2, p53 or scrambled control siRNA were either left untreated or treated with VP-16 and MG132 (20 μM) for 5 h, cells were then lysed and subjected to IP with MDM2 antibody or normal rabbit immunoglobulin (IgG), and then, IB was performed with the indicated Abs. **E**, **F** Confocal microscopy analysis of the colocalization of MDM2 and TOP2β. For 2 h, H1299 and A549 cells were exposed to VP-16 or not, and cells were subsequently harvested for immunofluorescence with the specified Abs. **G** MDM2 silencing extends the TOP2β protein half-life upon VP-16 treatment. H1299 cells were transfected with siCtrl or siMDM2 and treated with VP-16 and cycloheximide (CHX) for the indicated time points. Cells were harvested and lysed, and proteins were separated by SDS/PAGE and immunoblotted with the indicated Abs. **H** MDM2 but not p53 silencing extends the TOP2β protein half-life upon VP-16 treatment. A549 cells were transfected with siCtrl, siMDM2 or sip53 and treated with VP-16 and cycloheximide (CHX) for the indicated time points. Cells were harvested and lysed, and proteins were separated by SDS/PAGE and immunoblotted with the indicated Abs. **I** Depletion of MDM2 extends the protein half-life of TOP2β. Cells were transfected with sgRNA based on CRISPR‒Cas9-mediated knockout. Single-cell clones were sorted and then treated with CHX and VP-16 for various time periods, followed by IB with the indicated Abs. **J** MDM2 downregulation of TOP2β depends on its intact RING finger domain. After 48 h of transfection with the appropriate plasmids, HEK293 cells were subjected to 100 μg/ml CHX and 200 μM VP-16 doses for the times specified, followed by IB with the specified Abs. **K** Wild-type MDM2, but not the MDM2-C464A mutant, promotes TOP2β polyubiquitination. HEK293 cells were transfected with the indicated plasmids for 48 h and then treated with 20 μM MG132 and VP-16 for 5 h, followed by IP with HA beads and IB with the indicated Abs. All experiments were independently repeated three times. Densitometry quantification and fluorescence quantification was performed with Image J, and the quantification results are shown. *p < 0.05; **p < 0.01; ***p < 0.001; ^#^*p* < 0.05; ns no significant.

To further explore the mechanism by which MDM2 mediates the suppression of TOP2β, we then characterized the interaction between the two proteins. First, Flag-tagged MDM2 or Flag-tagged TOP2β was transfected into HEK293 cells. By using anti-Flag antibodies, we performed coimmunoprecipitation experiments 48 h after transfection and then analyzed the samples by western blot analysis. Notably, exogenous MDM2 was shown to interact with endogenic TOP2β, and exogenous TOP2β was able to interact with endogenic MDM2 (Fig. 2A, B). Moreover, this interaction was significantly augmented by VP-16 treatment. Next, to investigate the relationship between endogenous MDM2 and TOP2β, whole-cell lysates of H1299 and A549 cells were subjected to either an IgG control or anti-MDM2 antibody incubation, and antibodies against MDM2 and TOP2β were used to detect immunoprecipitants. We found that endogenous TOP2β coprecipitated with endogenous MDM2 from H1299 and A549 cell lysates but not with control IgG (Fig. 2C, D). In addition, VP-16 treatment boosted the interaction between TOP2β and MDM2 (Fig. 2C, D). Concurrently, as anticipated, the knockdown of p53 in A549 cells did not exert any discernible impact on the interaction between MDM2 and TOP2β (Fig. 2D). Finally, to further confirm the interaction between TOP2β and MDM2, immunofluorescence tests were executed in both H1299 and A549 cells. As expected, MDM2 and TOP2β were colocalized in the nucleus (Fig. 2E, F). Moreover, treatment with VP-16 in A549 cells significantly enhanced the fluorescence intensity of MDM2, indicating that there was a substantial increase in MDM2 protein levels (Fig. 2F).

Subsequently, we analyzed whether MDM2 binding to TOP2β decreased TOP2β protein levels by decreasing TOP2β stability. As illustrated in Fig. 2G–I, the knockdown of MDM2 by siRNA oligos or CRISPR‒Cas9-mediated MDM2 deletion greatly extended the protein half-life of TOP2β in H1299 and A549 cells cotreated with cycloheximide (CHX), an inhibitor of mammalian protein synthesis, and VP-16, suggesting that MDM2 enhances the degradation of TOP2β. Concomitantly, the knockdown of p53 in A549 cells does not alter the protein half-life of TOP2β (Fig. 2H). Besides p53, MDM2 has been shown to function as an E3 ubiquitin ligase, ubiquitinating and hence regulating the abundance of various target proteins. We subsequently examined whether or not MDM2’s RING finger and related ubiquitin ligase activity contributed to TOP2β degradation. An MDM2 RING finger mutant was created (MDM2-C464A), in which the cysteine was replaced with alanine, thus resulting in disruption of the integrity of MDM2’s RING finger and its E3 ligase activity. Immunoblotting assays revealed that ectopic expression of wild-type MDM2 (Flag-MDM2) facilitates the TOP2β degradation induced by VP-16 compared to empty-vector controls. In contrast, ectopic expression of MDM2-C464A inhibits TOP2β destruction and prolongs its half-life (Fig. 2J). Consistently, an in vivo ubiquitination assay confirmed that Flag-MDM2 greatly increased TOP2β polyubiquitination levels induced by VP-16 treatment, whereas the MDM2-C464A mutant significantly reduced the polyubiquitination of TOP2β (Fig. 2K), demonstrating that an intact RING finger domain and ubiquitin ligase function is necessary for MDM2 to downregulate TOP2β. Taken together, these findings collectively show that TOP2β and MDM2 directly interact and provide evidence that the MDM2 E3 ligase regulates the ubiquitination and destruction of TOP2β when subjected to VP-16 stimuli.

### RG7112 interferes with the association of MDM2 with TOP2β and blocks TOP2β degradation

**Fig. 3:**
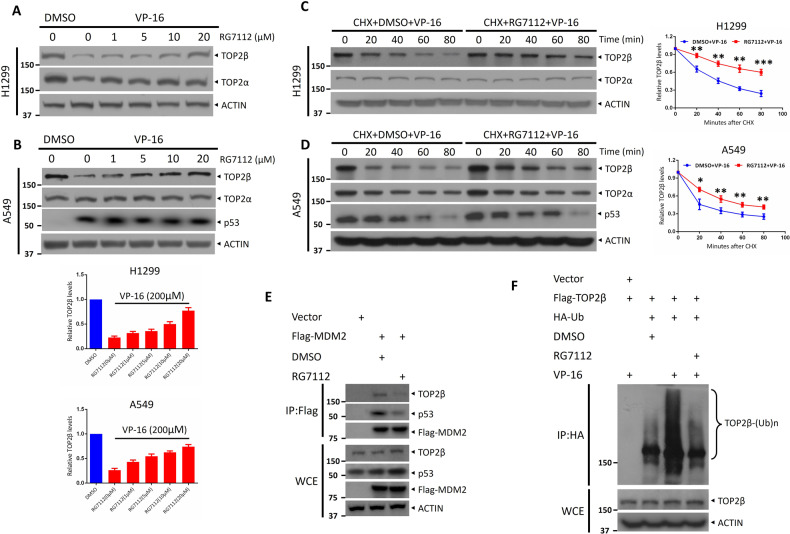
RG7112 interferes with the association of MDM2 with TOP2β and blocks TOP2β degradation. **A**, **B** RG7112 treatment dosage-dependently increased TOP2β levels. After incubating with VP-16 and RG7112 at different concentrations for 2 h, IB with the appropriate Abs was performed on H1299 and A549 cells. **C**, **D** RG7112 treatment prolonged the half-life of TOP2β protein after VP-16 treatment. H1299 and A549 cells were harvested at the indicated periods after administration of CHX and VP-16 or VP-16 and RG7112 (5 μM) and then subjected to IB with the appropriate Abs. **E** By administering RG7112, the physical interaction between MDM2 and TOP2β was inhibited. Following Flag-MDM2 transfection and 48 h of incubation with RG7112 or vehicle in HEK293 cells, IP with Flag beads and IB with the appropriate Abs were performed. **F** RG7112 treatment suppresses the polyubiquitination of TOP2β induced by VP-16 stimuli. HEK293 cells transfected with the specified plasmids were treated with VP-16 and MG132 (20 μM) or in association with RG7112 (5 μM) for 5 h, IP was then performed with anti-HA beads, and direct IB was performed with the selected antibodies. All experiments were independently repeated three times. Densitometry quantification was performed with Image J, and the quantification results are shown. *p < 0.05; **p < 0.01; ***p < 0.001.

RG7112 is a highly effective and specific MDM2 antagonist of the nutlin family that is currently in ongoing clinical trials [29]. To induce anti-cancer processes, RG7112 stabilizes p53. Mechanistically, to block the MDM2-p53 binding, RG7112 binds to the p53 receptor of MDM2, effectively masking the surface of MDM2 that binds to the substrate [30]. Thus, we investigated whether RG7112 could also inhibit MDM2 activity and prolong the half-life of TOP2β by interfering with TOP2β binding. We found that the reduction in TOP2β levels upon VP-16 stimulation was blocked by RG7112 in all tested cells (Fig. 3A, B). Moreover, the accumulation of TOP2β in all examined cells was dose-dependent after treatment with RG7112. (Fig. 3A, B). Similar to MDM2 knockout, TOP2β protein half-life was remarkably prolonged by RG7112 upon VP-16 stimulation (Fig. 3C, D). Furthermore, utilizing an IP-based pulldown experiment, it was discovered that when Flag-MDM2 was upregulated in HEK293 cells, RG7112 disrupted the interaction between Flag-MDM2 and endogenous p53 as well as TOP2β, with p53 serving as a positive control (Fig. 3E). Similarly, TOP2β polyubiquitination caused by VP-16 was greatly inhibited after RG7112 treatment (Fig. 3F). Altogether, our data demonstrate that RG7112 blocks TOP2β degradation by interfering with the association of MDM2 with TOP2β.

### Inactivation of MDM2 increases the level of TOP2β-DNA cleavage complex

**Fig. 4:**
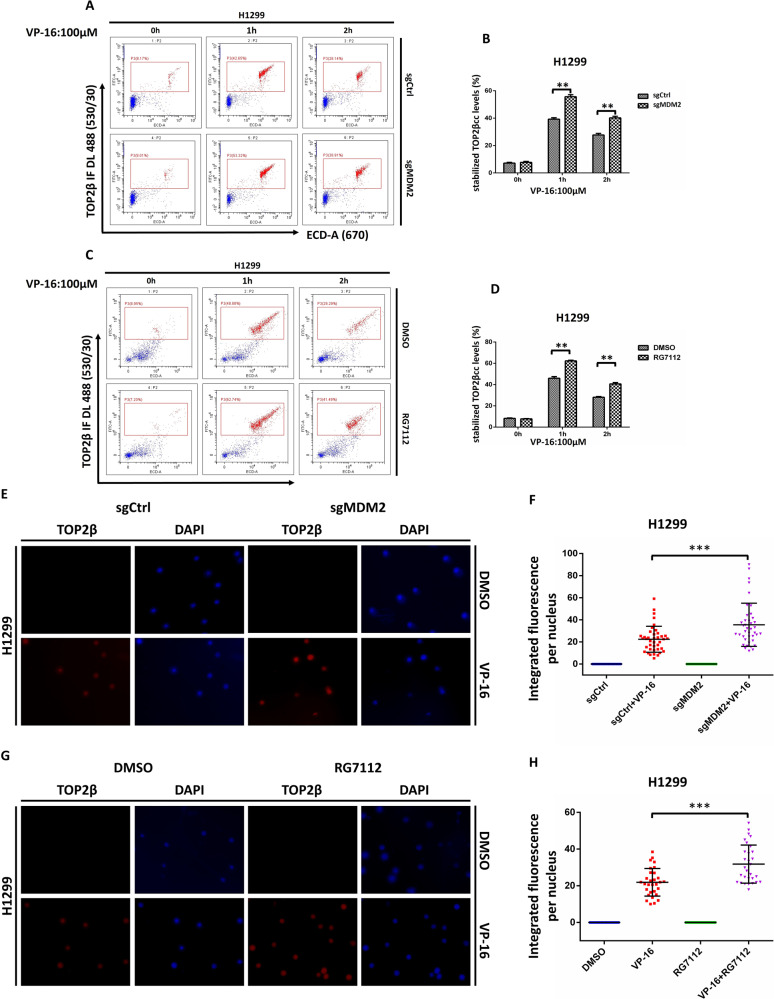
Inactivation of MDM2 increases the level of TOP2β-DNA cleavage complex. **A**, **B** MDM2 depletion occurs in a time-dependent manner, leading to an accumulation of TOP2βcc levels. H1299 cells were exposed to VP-16 for the durations specified, accompanied by fluorescence-activated cell sorting (FACS) tests to assess the levels of TOP2βcc. **C**, **D** The administration of RG7112 induced a time-dependent increase in TOP2βcc levels. TOP2βcc levels in H1299 cells were measured by FACS after administration of VP-16 monotherapy or in combination with RG7112. **E**–**H** Treatment with RG7112 or depletion of MDM2 augments the upregulation of TOP2βcc upon VP-16 exposure. VP-16 treatment was applied to H1299 cells (**E**, **F**) transfected with the specified sgRNA for 2 h. H1299 cells (**G**, **H**) with VP-16 or VP-16 and RG7112 (5 μM) for 2 h. The cells were then collected for the TARDIS assay. All experiments were independently repeated three times. Fluorescence quantification was performed with Image J, and the quantification results are shown. **p < 0.01; ***p < 0.001.

Previous studies have demonstrated that mammalian TOP2 forms at least two different complexes with DNA that are in rapid equilibrium: the TOP2-DNA cleavage complex and the non-cleavable complex [31]. To further confirm that MDM2 promotes the degradation of drug-stabilized TOP2β, which forms a cleavage complex with DNA, the TOP2β-DNA cleavage complex levels were measured via a flow cytometry-based method (TOP2cc-flow cytometry assay) established by Marcelo de Campos Nebel et al. [32]. As illustrated in Fig. 4A–D, VP-16 stimuli induce TOP2β to form cleavage complexes with DNA, while inactivation of MDM2 by genetic knockout or RG7112 treatment in H1299 cells induced a time-dependent increase in TOP2βcc levels.

Moreover, the levels of TOP2βcc were also assessed using the trapped in agarose DNA immunostaining (TARDIS) assay. In this assay, agarose was used to embed untreated or drug-treated cells on microscope slides. The cells are subsequently lysed to rupture the cellular membranes and extract soluble proteins, followed by salt filtration for nuclear protein removal and any DNA matrix-bound topoisomerase that is not covalently attached. The drug-stabilized TOP2-DNA complex was then identified using isoform-specific antisera and a secondary antibody labeled with Alexa Fluor 568. Fluorescence levels were assessed and quantified using digital images of DAPI fluorescence (DNA) and Alexa Fluor 568 immunofluorescence (drug-stabilized TOP2βcc). As expected, the levels of remaining stabilized TOP2βcc were increased by MDM2 knockout or RG7112 treatment, as reflected by the higher levels of fluorescence intensity (Fig. 4E–H). Our results collectively support the concept that MDM2 promotes the degradation of drug-stabilized TOP2β, thereby reducing the level of TOP2βcc.

### MDM2 is involved in ICRF-193-induced TOP2β degradation

**Fig. 5:**
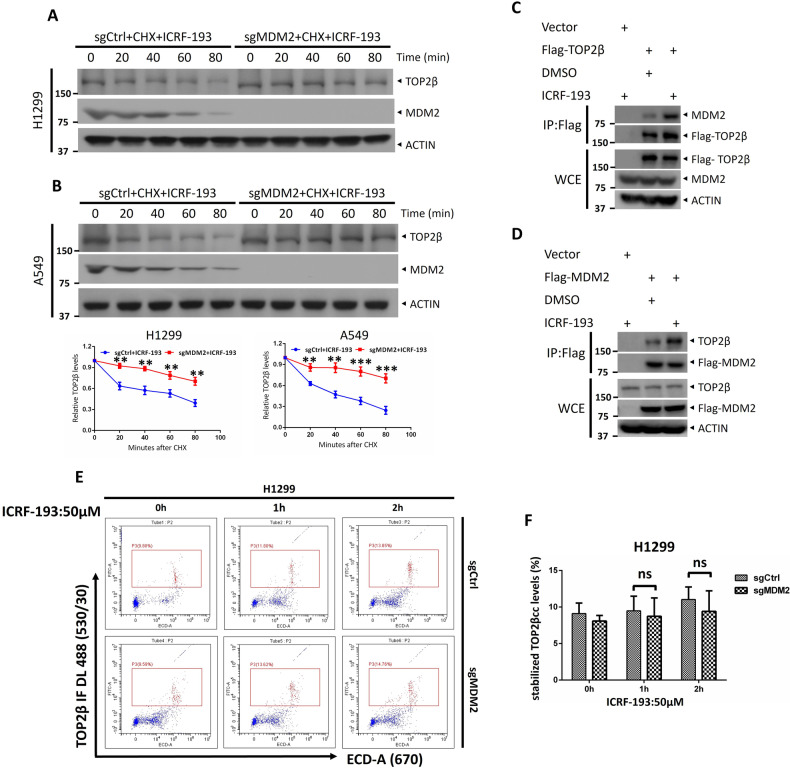
MDM2 is involved in ICRF-193-induced TOP2β degradation. **A**, **B** Depletion of MDM2 extends the TOP2β protein’s half-life after ICRF-193 treatment. Following the transfection of H1299 (**A**) and A549 (**B**) cells with sgRNA based on CRISPR‒Cas9-mediated knockout, they were treated with ICRF-193 and cycloheximide (CHX) for the specified time intervals. IB with the specified Abs was performed on the collected cells. **C**, **D** ICRF-193 treatment increased the interaction between TOP2β and MDM2. After 48 h of transfection, HEK293 cells were incubated with ICRF-193 (50 μM) and MG132 (20 μM) for 5 h, and then subjected to IP with Flag beads and IB with the indicated Abs. **E**, **F** MDM2 inactivation did not alter the basal level of TOP2βcc. After treatment of H1299 cells with ICRF-193 for the respective periods, the levels of TOP2βcc were determined by FACS. ns no significance. All experiments were independently repeated three times. Densitometry quantification was performed with Image J, and the quantification results are shown. **p < 0.01; ***p < 0.001; ns no significant.

It has been well established that TOP2 poisons inhibit the DNA re-ligation function of TOP2, thus stabilizing TOP2βcc, which contains TOP2β-concealed DSBs. It will be intriguing to explore whether the initial trigger for TOP2β degradation is driven by concealed DSBs and whether MDM2 participates in the maintenance of the levels of the non-cleavable TOP2β-DNA complex. In the current investigation, we utilized the TOP2 catalytic inhibitor ICRF-193, which does not capture the TOP2–DNA covalent complex but stabilizes ATP-bound TOP2β in the closed-clamp conformation without forming a DNA lesion [33]. Figure 5A, B demonstrates that ICRF-193 triggers the degradation of TOP2β, while MDM2 knockout also remarkably lengthens the half-life of the TOP2β protein. Next, we determined whether ICRF-193 could affect the interaction between MDM2 and TOP2β. Indeed, a pull-down assay revealed that Flag-TOP2β interaction with endogenous MDM2 (Fig. 5C). Moreover, a coimmunoprecipitation test using ectopically expressed Flag-MDM2 verified the interaction between MDM2 and TOP2β (Fig. 5D). Additionally, treatment with ICRF-193 greatly increased this interaction (Fig. 5C, D). Finally, a TOP2cc-flow cytometry assay confirmed that ICRF-193 does not induce the formation of TOP2βcc, and MDM2 inactivation did not alter the basal level of TOP2βcc (Fig. 5E, F). Collectively, these data imply that MDM2-mediated TOP2β degradation triggered by the TOP2β-DNA covalent complex is not due to DNA damage and that MDM2 is also responsible for the elimination of TOP2β in the closed-clamp conformation.

### Inactivation of MDM2 impairs the DNA damage response by concealing VP-16-induced DSBs

**Fig. 6:**
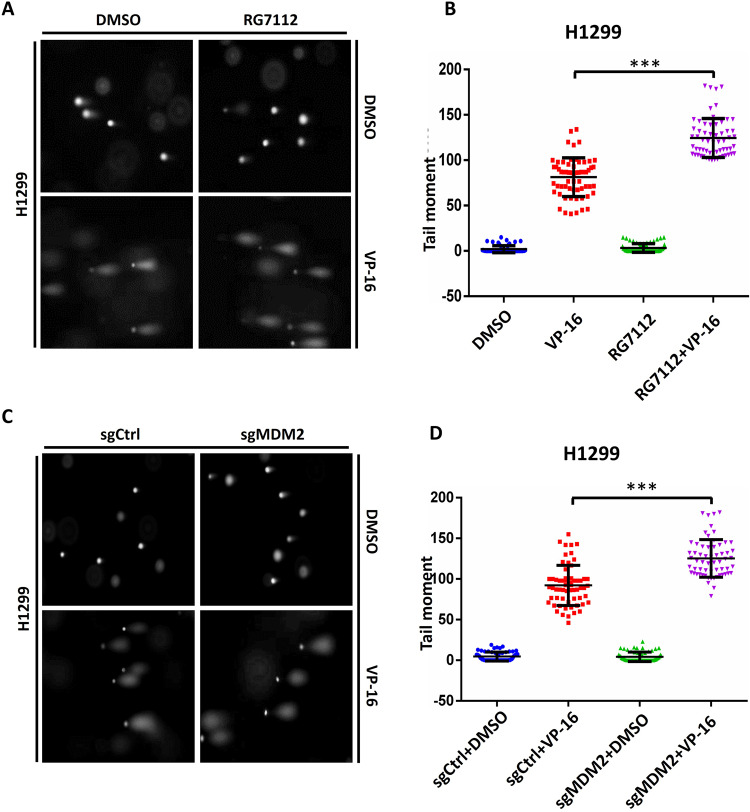
Inactivation of MDM2 impairs the DNA damage response by concealing VP-16-induced DSBs. **A**, **B** RG7112 treatment enhances VP-16-induced DSBs. For 1 h, H11299 cells were pretreated with RG7112 (5 μM), followed by 2 h of cotreatment with VP-16. **C**, **D** Depletion of MDM2 enhances VP-16-induced DSBs. H1299 cells transfected with the indicated sgRNA were treated with VP-16 for 2 h. Cells were then harvested for the neutral comet assay. Comet tail moments were analyzed from at least 50 cells for each experimental condition, and the data are presented as the mean ± SEM from three independent experiments, ***p < 0.001.

It has been reported that the stabilization of TOP2cc by TOP2 poisons permits cellular processes to convert TOP2-concealed DNA strand breaks into true DSBs, which provokes a DNA damage response [34]. The neutral comet assay was employed to measure the level of DSBs in the presence of RG7112 to inhibit the activation of MDM2 after VP-16 treatment to determine whether MDM2-mediated TOP2β degradation is a key factor in the conversion of TOP2βcc into true DSBs that are recognized by DNA damage signaling. Noticeably, the comet tail moment identified by the neutral comet assay mirrored the overall abundance of TOP2 concealed DSBs and protein-free DSBs. Moreover, protease K was used in the lysis solution to digest TOP2β in TOP2βcc to reveal DSBs concealed by TOP2β. As expected, treating H1299 cells with VP-16 considerably increased the comet tail moment, demonstrating that VP-16 caused DSBs, a result that is in line with the notion that VP-16 is involved in maintaining TOP2-DNA covalent complexes to induce DSBs. Furthermore, compared with VP-16 monotherapy, cotreatment with RG7112, which hindered TOP2β degradation, resulted in a substantial increase in the number of DSBs, as evaluated by the percentage of tail DNA after treatment (Fig. 6A, B). Additionally, comparable outcomes were observed when MDM2 was selectively deactivated by CRISPR‒Cas9-mediated MDM2 knockout (Fig. 6C, D). Most recently, it was found that cancer cell lines that overexpress MDM2 show attenuated levels of γH2AX, a key mediator of DNA damage signaling, as a result of VP-16 treatment, while treatment with neocarzinostatin (a genotoxic anti-cancer drug) did not result in a noticeable difference in γH2AX [11]. These results suggest that MDM2 can mediate cancer cells with selective resistance to DNA DSBs induced by TOP2 poison. In conclusion, the data presented above show that MDM2 ubiquitin ligase inactivation prevents destruction of TOP2β induced by VP-16 therapy, conceals DSBs, and maintains TOP2β-DNA covalent complexes, leading to an elevation in DSBs as measured by the neutral comet test.

### RG7112 makes cancer cells more susceptible to the effects of VP-16 by triggering the apoptotic process

Existing literature has shown that TOP2 poisons prevent TOP2 from performing its ligation function, leading to the stable creation of TOP2cc, in which the TOP2 protein remains covalently associated with the DNA, which contains TOP2-concealed DSBs. Stable TOP2cc is a fundamental component of genome maintenance, and the importance of TOP2cc is compounded by its essential function in mediating the cytotoxicity of TOP2 poisons. Malignant cells, however, have evolved strategies for removing TOP2 from TOP2-DNA adducts in order to repair DSBs induced by TOP2 poisons, thereby exposing TOP2 concealed DNA DSBs and activating the DDR, which is also a major threat to the survival of the cells. Therefore, it is yet unknown if the degradation of TOP2β caused by these anti-cancer medications contributes to the death of cancer cells.

**Fig. 7:**
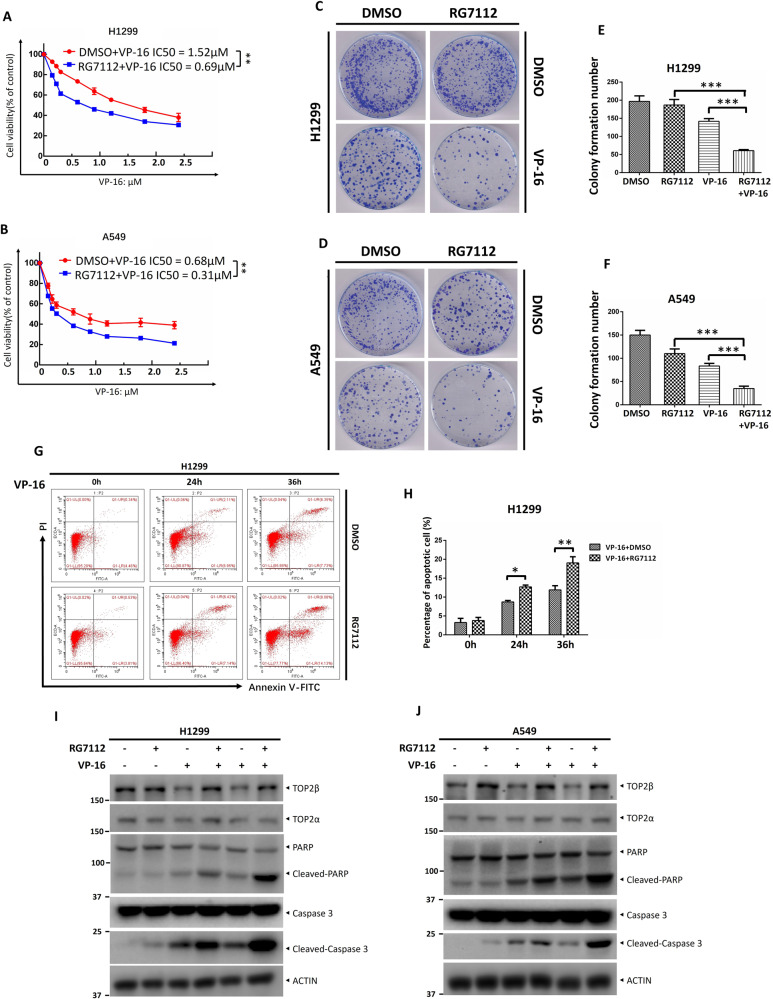
RG7112 makes cancer cells more susceptible to the effects of VP-16 by triggering the apoptotic process. **A**, **B** RG7112 sensitizes cells to VP-16 treatment. H1299 and A549 cells were treated with various concentrations of VP-16 alone or in combination with an IC20 concentration of RG7112 and then subjected to an ATP-lite assay. **C**–**F** RG7112 enhances the cytotoxicity in lung cancer cells by VP-16 treatment. Cells were plated in triplicate in 60-mm dishes and treated with various concentrations of VP-16 alone or in combination with RG7112. After 10–14 days, colonies were stained and counted (>50 cells in a colony). **G**, **H** RG7112 administration enhances apoptosis induced by VP-16 treatment. Cells were treated with VP-16 alone or in combination with RG7112. for the indicated time periods, the cells were harvested and subjected to FACS analysis to determine the apoptotic population. **I**, **J** RG7112 administration enhances apoptotic activity triggered by VP-16 treatment. Cells were incubated with VP-16, followed by RG7112 or DMSO for 36 h. Cells were harvested for IB with the indicated antibodies. All experiments were independently repeated three times. Densitometry quantification was performed with Image J, and the quantification results are shown. *p < 0.05; **p < 0.01; ***p < 0.001.

As mentioned above, RG7112 is an effective inhibitor of MDM2 that perfectly fills the three main binding pockets of MDM2 and blocks MDM2 attachment to TOP2β, leading to TOP2β stabilization and accumulation of TOP2cc. Next, we explored whether RG7112 administration would improve cancer cell eradication caused by VP-16 treatment. To begin, we tested a range of RG7112 concentrations on H1299 and A549 cells to ascertain their respective IC20 values. The ATPlite cell viability assay proved that p53-null H1299 cells have incredible RG7112 resistance, with an IC_20_ of 8.1 μΜ, followed by p53 wild-type A549 cells with an IC_20_ of 0.08 μΜ (Fig. S1a, b). Then, we calculated the IC_50_ values of VP-16 administration with and without RG7112 based on the IC_20_ concentration of RG7112 in conjunction with different doses of VP-16. The ATPlite assay demonstrated that cell viability was reduced as the VP-16 concentration increased, indicating its anti-cancer efficacy. The combination group (RG7112 + VP-16) experienced a greater decline in cell viability relative to the VP-16-only group. The combination group’s IC50 values dropped remarkably, from about 1.52 μM to 0.69 μM in H1299 cells and from around 0.68 μM to 0.31 μM in A549 cells (Fig. 7A, B). These results indicated that RG7112 can sensitize lung cancer cells to VP-16 treatment and is not dependent on p53 status. In addition, colony formation was much lower in the RG7112 + VP-16 group than in the RG7112 or VP-16 monotherapy groups, confirming that the combination substantially lowered H1299 and A549 cell survival (Fig. 7C–F). Apoptosis is a well-known mechanism for VP-16-induced suppression of cell growth. Next, we tested if RG7112 may synergistically enhance the apoptotic effects of VP-16 on cells. To begin, H1299 cells were exposed to VP-16 at varying doses, with or without RG7112. Next, flow cytometric analysis of Annexin V+ cells revealed the following: (1) VP-16 treatment alone promoted cancer cell apoptosis to a certain extent, and (2) a significantly greater apoptotic rate was triggered in the combination group compared to the VP-16 monotherapy group, as shown by the observation that there is a remarkably higher proportion of Annexin V+ population in the combination group (Fig. 7G, H). Likewise, the combination of VP-16 and RG7112 significantly increased caspase-3 and PARP cleavage (Fig. 7I, J), two characteristics of apoptosis [35], relative to those of VP-16 or RG7112 treatment alone, suggesting that RG7112 administration sensitizes cancer cells to VP-16 treatment by inducing apoptosis. Overall, our data demonstrate that MDM2 protects cells against TOP2 poisons by directing ubiquitin and proteasome disintegration of TOP2β. Therefore, blocking MDM2-mediated TOP2β degradation by RG7112 synergistically suppresses lung cancer cell survival following treatment with VP-16, resulting in an increase in apoptosis and susceptibility to VP-16 treatment.

### Combination of RG7112 with VP-16 synergistically increased inhibition of tumor growth in BALB/c xenograft tumor model and in situ lung cancer mouse model

**Fig. 8:**
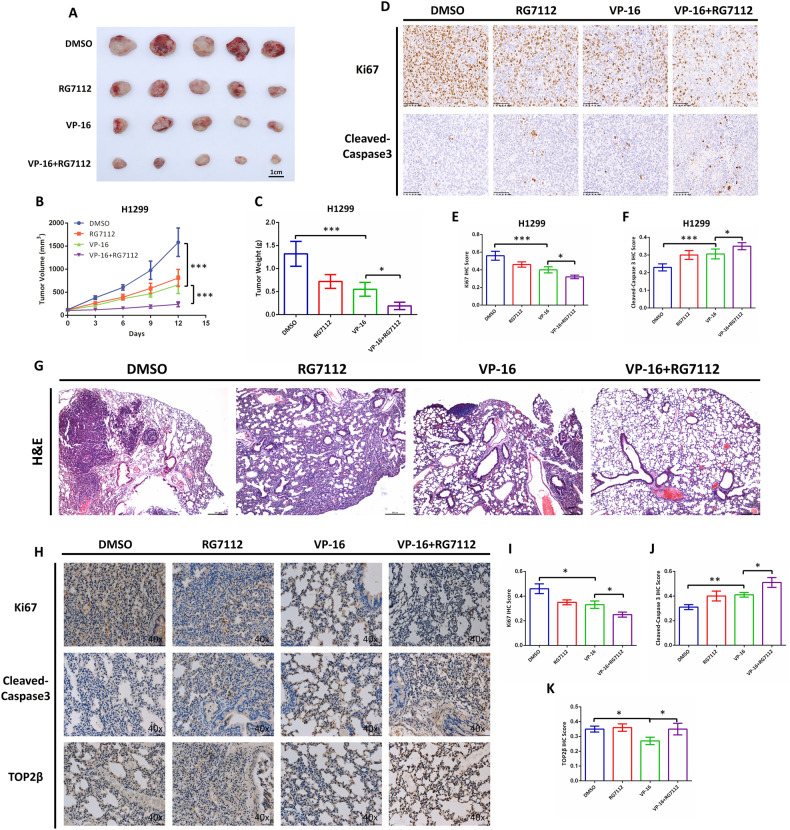
Combination of RG7112 with VP-16 synergistically increased inhibition of tumor growth in BALB/c xenograft tumor model and in situ lung cancer mouse model. **A**–**C** H1299 cells were implanted subcutaneously into BALB/c nude mice. When the tumor volume reached approximately 90–110 mm^3^, four mice groups were established at random and injected with different drugs intravenously as described in Materials and Methods. **A** Representative images of each treatment group are shown. **B** A Vernier caliper was utilized to evaluate the changes in tumor volume. **C** After receiving treatment, the mice categorized into groups had their tumor weights compared. **D**–**F** Immunohistochemical analysis of Ki-67 and Cleaved-Caspase 3 was performed in tumor tissues under a bright field microscope. **D** Representative images of each treatment group are shown. The quantification of Ki-67 IHC score (**E**) and Cleaved-Caspase 3 IHC score (**F**) in each group was shown in the histogram. **G**–**K** An in-situ lung cancer mouse model was generated by percutaneous injection of H1299 cells into C57BL/6 mice’s right lung. Two weeks later, different drugs were administered intravenously to mice that were randomized into four groups. After a further two weeks, animals were euthanized and their lung tissue were collected and stained with H&E or the indicated antibodies (as detailed in the Materials and Methods section). **G** Representative photographs of H&E staining. **H** Representative images of Ki-67, Cleaved-Caspase 3, and TOP2β Immunohistochemical analysis. The quantification of Ki-67 IHC score (**I**), Cleaved-Caspase 3 IHC score (**J**), and TOP2β IHC score (**K**) in each group was shown in the histogram. *p < 0.05; **p < 0.01; ***p < 0.001.

Inspired by the in vitro results, we then evaluated the efficacy of the combinatorial therapy in a preclinical mouse model bearing H1299 tumor xenografts. Specifically, the model was created by implanting H1299 cancer cells subcutaneously into immunodeficient BALB/c nude mice. After the tumor volume in the mice attained approximately 90–110 mm^3^, the mice were randomized into four groups, and each group received a different drug through intravenous injection. As illustrated in Fig. 8A–C, the relative tumor volume and the relative tumor weight in the combination group (RG7112 + VP-16) were significantly reduced when compared with the VP-16-only or RG7112-only group. Additional, histopathological analyses of the excised tumors were further performed. Consistent with the growth kinetics, more extensive intra-tumoral apoptosis was induced by the concomitant administration of RG7112 and VP-16, which correlated well with the reduction in Ki-67 proliferation (Fig. 8D–F).

Furthermore, we generated an in-situ lung cancer mouse model by percutaneous injection of H1299 cells into the right lung of C57BL/6 mice. Two weeks later, the mice were randomized into four groups, and each group received a different medication administered intravenously. After another two weeks, mice were euthanized and the lungs tissue were collected, along with subsequent staining with hematoxylin and eosin (H&E) or the indicated antibodies. Notably, H&E staining analysis revealed that the mice treated with the combinatorial regimens (RG7112 + VP-16) exhibited remarkably decreased lung tumor burden when compared with VP-16-only or RG7112-only group (Fig. 8G). Furthermore, as shown in Fig. 8H–K, Immunohistochemistry (IHC) staining with the indicator Ki-67 and Cleaved-Caspase 3 validated the retarded cell proliferation and more extensive intra-tumoral apoptosis in excised lung tissues after combinatorial regimens (RG7112 + VP-16) therapy. Moreover, single-agent treatment of VP-16 significantly decreased the levels of TOP2β. Consistent with the in vitro results, the combinatorial therapy using RG7112 and VP-16 rescued the TOP2β levels. In summation, these in vivo data corroborate the notion that the combination of MDM2 inhibitors and TOP2β poisons has excellent in vivo anti-cancer efficacy.

## Materials and methods

### Cell lines and chemicals

The H1299, A549, and HEK293 cell lines were all procured from the American Type Culture Collection (ATCC), and preserved in Dulbecco’s modified Eagle’s medium (DMEM) containing 10% (v/v) fetal bovine serum (FBS) and 1% penicillin/streptomycin. CRISPR-Cas9 knockout of MDM2 was achieved in H1299 and A549 cells after being transfected with a sequence-verified CRISPR plasmid and puromycin (S7417; Selleck) selection. All cell lines were grown in a 37 °C, 5% CO2 incubator. Commercial suppliers provided the following chemicals: DMSO (Sigma), VP-16 (MCE), CHX (Sigma), ICRF-193 (Sigma), and RG7112 (MCE).

### Immunoblotting and immunoprecipitation

Following the lysis of cells in a lysis solution containing protease and phosphatase inhibitors, the cells were subjected to ultrasound. After the ultrasound, the supernatant was harvested, and the concentration of protein was calculated using the BCA protein assay kit. Immunoblotting (IB) or immunoprecipitation (IP) assay was performed as reported previously [10]. The following antibodies (Abs) were used: MDM2 (OP46, Calbiochem, 1:1000), MDM2 (86934 S, Cell Signaling Technology, 1:500), TOP2β (611493, BD Biosciences, 1:1000), TOP2β (sc-25330, Santa Cruz, 1:1000), TOP2α (12286, Cell Signaling Technology, 1:1000), p53 (2527 S, Cell Signaling Technology, 1:1000), p21 (2947 S, Cell Signaling Technology, 1:1000), p-CHK1 (2348, Cell Signaling Technology, 1:2000), CHK1 (sc-8408, Santa Cruz, 1:1000), ACTIN (A5441, Sigma, 1:10000), β-TrCP1 (4394, Cell Signaling Technology, 1:1000), FLAG (F1804, Sigma, 1:2000), γH2AX (05-636, Millipore, 1:1000), PARP (9542, Cell Signaling Technology, 1:1000), Caspase 3 (9665, Cell Signaling Technology, 1:1000), HA (11867423001; Roche; 1:2000).

### Quantitative RT-PCR

To extract total RNA, we used TRIzol reagent (15596018; Invitrogen) following the kit guidelines before transcribing it into cDNA utilizing the PrimeScript RT reagent kit (RR037A; Takara). On an Applied Biosystems StepOnePlusTM Real-Time PCR equipment, expression levels were evaluated via Quantitative real-time PCR (qRT-PCR) with the SYBR® Premix Ex TaqTM kit (Tli RNaseH Plus; Takara Biotechnology) as directed by the manufacturer. qRT-PCR primers used were as follows: 5′-GGC ATT CCA AAG AAG ACT ACA ACA CCA-3′ and 5′-TAC TTC TTT CCT AGC CCG ACC GGT-3′ for TOP2β; 5′-CCC CAA CTT TGA TGT GCG TG -3′ and 5′-TCC ATG TTC TGA CGG GAA GC-3′ for TOP2α; 5′-CCG GAT CTT GAT GCT GGT GT-3′ and 5′-ATC ACT CTC CCC TGC CTG AT-3′ for MDM2; 5′-AGG GCA TCC TGG GCT ACA C-3′ and 5′-GCC AAA TTC GTT GTC ATA CCA G-3′ for GAPDH.

### siRNA silencing and CRISPR/Cas9-mediated knockout

The following plasmids or siRNA oligos were transfected into sub-confluent cells utilizing Lipofectamine 3000 (Invitrogen) per the directions stipulated by the manufacturer. The following sequences of siRNA oligos were used: siCtrl: 5′-ATT GTA TGC GAT CGC AGA C-3′; siMDM2: 5′-GCC AGT ATA TTA TGA CTA A -3′; sip53: 5′-CAC CAT CCA CTA CAA CTA CAT-3′.

To knock out MDM2, single-guide RNA (sgRNA) was subcloned into pSpCas9(BB)-2A-Puro plasmid (PX459). H1299 and A549 Cells were transfected with the construct and chosen with puromycin for three days. Cells were then plated at a low density and single clones were selected using a microscope. The loss of MDM2 was validated by sequencing and immunoblotting. The sequence of MDM2-sgRNA:5′ -GTT GGG CCC TTC GTG AGA AT-3′.

### In vivo ubiquitination assay

As per the guidelines provided by the manufacturer, PolyJet (SL100688; SignaGen Laboratories) was utilized for plasmid transfection into HEK293 cells. After 48 h, cells were pretreated using VP-16 or RG7112 in conjunction with MG132 for the stated durations. Following cell lysis in a buffer containing protease and phosphatase inhibitors, the cells were incubated with HA agarose beads, as previously described [10]. Finally, the beads were rinsed four times with lysis buffer, boiled, and then subjected to direct IB with anti-TOP2 or anti-FLAG antibody.

### Immunofluorescence

Cells were grown on a coverslip treated with VP-16 or DMSO. After treatment, paraformaldehyde (PFA) at 4% was utilized to fix the cells for 15 min, followed by permeabilization in 0.3% Triton X-100 for 25 min and blocking for 1 h with goat serum. Anti-MDM2 and anti-TOP2β antibodies were then used to stain cells at room temperature for 2 h. Finally, MDM2 and TOP2β were labeled with species-specific fluorescein-conjugated secondary antibodies Alexa Fluor 568 donkey anti-mouse IgG (H + L) and Alexa Fluor 488 donkey anti-rabbit IgG (H + L). Next, DAPI was used to counterstain DNA. A fluorescent microscope was used to view and capture images of the cells.

### TOP2cc-flow cytometry assay

TOP2cc-flow cytometry assays were performed following previous procedures [32]. Briefly, cells were treated for varying times with vehicle or VP-16. Then, the cells were washed and resuspended in PHEM buffer containing 2 mM PMSF. Next, the cells were fixed in 4% paraformaldehyde solution, and proteins were extracted by adding extraction buffer. Finally, following blocking, the cells were labeled with anti-TOP2β antibody. The TOP2βcc and DNA were then labeled with the appropriate fluorescein-conjugated secondary antibody and propidium iodide. The TOP2βcc levels were measured and analyzed by flow cytometry.

### TARDIS assay

TOP2 intermediates on genomic DNA were generated by etoposide-treated cells. After being treated, cells are immobilised on a glass slide using agarose. After washing and extracting the slides with SDS and salt, the majority of cellular components are eliminated, but genomic DNA and covalent adducts remain. Adducts were subsequently labeled utilizing a polyclonal antibody against TOP2β and an Alexa Fluor 568-conjugated secondary antibody. The slides were then stained for 15 min with DAPI and cover slips were put and fastened. Then, images of blue (DAPI-stained DNA) and red (Alexa Fluor 568-stained covalently bound TOP2) immunofluorescence were taken using an epifluorescence microscope equipped with a slow scan charge-coupled camera. TARDIS analysis measured the TOP2β-DNA cleavage complex as reported previously [36].

### Neutral comet assay

The neutral comet assays were conducted in the same manner as detailed previously [37]. Succinctly, cells on 60-mm plates were exposed to 2 mM thymidine. After 24 h, the cells were rinsed with PBS and treated with VP-16 or RG7112 alone or in combination for an additional 1 h. The collected cells were then placed on the slide. Cells were then lysed by submerging the slides at 37 °C for a whole night in a neutral N1 lysis solution (2% sarkosyl, 0.5 M EDTA, 0.5 mg/ml proteinase K, pH 8.0). Then, propidium iodide (PI) dye at 10 μg/ml was used to stain the slides for 20 min, and the cells were subjected to a 25-minute (0.6 V/cm) electrophoresis at 15 V before being analyzed by fluorescence microscopy. The software package CometScore was utilized to examine the comet tail moment.

### Cell viability and clonogenic survival assays

H1299 and A549 cells were plated in 96-well plates in triplicate at a density of 2000 to 3000 cells per well. These cells were subsequently treated for 72 h with VP-16 alone or in combination with RG7112 at various doses. The ATPlite assay (6016731; Perkin-Elmer) was utilized to test the viability of the cells according to the guidelines provided by the manufacturer. The results from three independent, triplicate-run tests are shown.

Cells were cultured on 0.1% gelatin-coated 60-mm plates (Sigma, V900863) for clonogenic survival studies, pretreated with the specified treatments for 1 h, and then grown in new media. After 10 to 14 days, the cells were dyed for 20 min with Coomassie brilliant blue solution, followed by a wash under running water. Following air-drying of the cell colonies, they were photographed for colony counting. The data from three separate studies are presented as mean ± SEM.

### Animal experiments

For the xenograft experiment, BALB/c nude mice were first injected subcutaneously with 3*10^6 of H1299 cells. Tumors were allowed to grow for two weeks until their volume reached 90–110 mm^3^. The mice were further divided into four groups at random (*n* = 5), and intravenously injected with vehicle (5% DMSO + 30% PEG 300 + 5% Tween 80 + ddH2O), RG7112 (25 mg/kg, in vehicle), VP-16 (20 mg/kg, in vehicle) or RG7112 + VP-16 (25 mg/kg + 20 mg/kg, in vehicle) three times on days 0, 3 and 6. Every three days, we weighed and measured the mice to obtain their body weights and tumor volumes. When the control mice reached humane endpoints, all of the animals were euthanized and their tumors were immediately weighed and photographed. The equation below was used to calculate tumor volumes: length × width^2^ × 0.5.

For the in-situ lung cancer mouse model, the C57BL/6 mice were placed into a dorsal decubitus posture after being anesthetized using a respiratory anesthesia machine. Under radiographic control, 1*10^6 H1299 cells in a solution containing 50 μl of PBS and 50 μl of mouse sarcoma extracellular matrix (Matrigel, BD Biosciences, NJ, USA) were injected percutaneously into the right lung of the animals using 1-mL tuberculin syringes with 30 G hypodermic needles (Becton Dickinson, NJ, USA). The animals were subsequently placed on a heating pad to reset till they had completely recovered. Two weeks later, mice were randomly divided into four groups and intravenously injected with vehicle (5% DMSO + 30% PEG 300 + 5% Tween 80 + ddH2O), RG7112 (25 mg/kg, in vehicle), VP-16 (20 mg/kg, in vehicle) or RG7112 + VP-16 (25 mg/kg + 20 mg/kg, in vehicle) three times on days 0, 3 and 6. Continuous observation was carried out on the mice once every three days. After another two weeks, all animals were sacrificed and the lung tissues were collected and stained with H&E or the indicated antibodies.

BALB/c nude mice (male; body weight, 18–22 g; 6–8 weeks old) and C57BL/6 mice (male; body weight, 18–22 g; 6 weeks old) were purchased from Shanghai Slack Laboratory Animals Co., Ltd. (Shanghai, China). Animal study was approved by the Institutional Ethics Committee of Ningbo University. Animal care was provided in accordance with the principles and procedures outlined in the Chinese National Research Guide for the Care and Use of Laboratory Animals.

### Immunohistochemistry (IHC)

Succinctly, 5 μm thick sections of tumor tissue or mouse lung tissues were stained with hematoxylin and eosin or the antibodies, as previously described [38, 39]. The following antibodies were used: Ki-67 (9027, Cell Signaling Technology, 1:200), Cleaved Caspase-3 (9661, Cell Signaling Technology, 1:400), TOP2β (20549-1-AP, Proteintech, 1:100). The slides were then scanned using an Aperio Whole Slide Scanner. The quantitative evaluation for expression was determined as previously described [38, 39].

### Statistical analysis

To compare parameters between two groups, Statistical Program for the Social Sciences 20.0 (SPSS, Chicago, IL, USA) was employed to perform a two-tailed Student’s *t* test for statistical analysis. One-way ANOVA followed by Tukey’s post hoc test was used to compare >2 groups. Tumor proliferation analysis was performed using two-way repeated measures ANOVA. The data from three different tests are shown as the mean ± SD or mean ± SEM. *p* < 0.05 was found to be statistically significant.

## Discussion

Multiple investigations have shown that TOP2 poisons trigger TOP2β degradation via the 26 S proteasome [9, 40]. Recently, our group demonstrated that the SCF^β-TrCP^ ubiquitin ligase contributes to the degradation of TOP2β caused by TOP2 poisons [10], whereas β-TrCP knockout only partially suppresses the polyubiquitination of TOP2β. These findings imply that there may be other unidentified pathways that control TOP2β stability. In this study, we identified and characterized MDM2 as the E3 ubiquitin ligase for the TOP2β protein by the following lines of evidence: (1) MDM2 silencing significantly inhibits the TOP2β downregulation triggered by VP-16; (2) MDM2 and TOP2β directly interact and both colocalize in the nucleus; (3) inactivation of MDM2 causes the accumulation of TOP2β by extending its protein half-life; (4) MDM2 enhances TOP2β polyubiquitination together with the destruction of TOP2β, a process that depends on its intact RING finger domain; and (5) RG7112, an MDM2 inhibitor, causes TOP2β to accumulate dosage dependently and extends the TOP2β half-life by disrupting MDM2 binding to TOP2β and reduces TOP2β polyubiquitination. Additionally, TOP2 poisons block the re-ligation function of TOP2, resulting in the formation of TOP2cc, which contains TOP2-concealed DSBs. Next, we demonstrated that MDM2 promotes the VP-16-induced degradation of TOP2β, thereby converting TOP2βcc into true DSBs and initiating DNA damage signaling, as supported by the following evidence: (1) inactivation of MDM2 by genetic knockout or RG7112 treatment in H1299 cells increased the levels of stabilized TOP2βcc and triggered the time-dependent accumulation of TOP2βcc; and (2) Upon VP-16 treatment, MDM2 deletion or RG7112 treatment resulted in an increase in DSBs in the neutral comet experiment. Biologically, we established that MDM2-mediated TOP2β degradation increases cancer cell survival, since its inhibition by RG7112 decreases the survival of lung cancer cells synergistically with VP-16 treatment, resulting in increased apoptosis and higher sensitivity to VP-16 treatment (Fig. 7).

TOP2α and TOP2β are two isozymes found in human cells; they share approximately 72% sequence identity [41]. However, they are regulated quite differently, and TOP2β is preferentially degraded over TOP2α in response to TOP2 poison stimuli [40, 42]. Indeed, we found that VP-16 treatment induced a substantial degradation of TOP2β while having little, if any, influence on TOP2α (Fig. 1A, B). Additionally, the protein level of TOP2α peaks at the G2/M phase and performs a critical function in the survival of proliferative and malignant cells [43]. Nevertheless, the level of TOP2β protein is not remarkably altered throughout the cell cycle and is not necessary at the cellular level [43]. Furthermore, TOP2α was discovered to be colocalized with DNA replication sites [44], suggesting that TOP2α is crucial for DNA replication and chromosome condensation, whereas TOP2β was localized in the rRNA-encoding DNA’s transcribed region and may be more critical for transcription [45, 46]. Consistent with this finding, TOP2β-induced DNA strand breaks are enriched at transcriptional start sites proportional to the extent of transcription [47]. Importantly, the TOP2βcc bound to DNA strands prevents the Pol transcription-elongation complex from moving [48]. Arrest transcription then induces 26 S proteasome-dependent destruction of TOP2β [48]. The signal responsible for the destruction of TOP2β following the blocking of the Pol elongation complex should be investigated in the future. Collectively, it seems that preferential destruction of TOP2β by TOP2 poisons could be correlated with the interaction of TOP2βcc and the Pol elongation complex, while TOP2αcc is rarely encountered at replication forks because the RNA polymerase transcription elongation complex is significantly more likely to encounter protein blocks and DNA lesions than replicative DNA polymerase along the same DNA template [49].

Existing literature illustrates that TOP2 poisons inhibit the DNA religation function of TOP2, thus stabilizing TOP2βcc, which contains TOP2β-containing DSBs. However, it is unknown whether the initial trigger for TOP2β downregulation mediated by MDM2 is specifically induced by concealed DSBs. In fact, we observed that MDM2 is also responsible for the elimination of TOP2β induced by ICRF-193, a TOP2 catalytic inhibitor that does not generate a DNA damage response, but traps TOP2 in a circular clamp, indicating that the MDM2-mediated destruction of TOP2β induced by the TOP2β-DNA covalent complex is not related to DNA damage (Fig. 5).

DNA double-strand breakage can be induced by many endogenous and exogenous factors, such as ionizing radiation, chemicals, and physical inducers. Among all known types of DSBs, TOP2 poisons and TOP2-derived DSBs are unique in that the tyrosine residues of the degraded TOP2 are covalently linked to the 5’ end of the fractured DNA strand via a phosphodiester bond [50]. Hence, the repair of TOP2-based DSBs requires the removal of these covalent protein‒DNA crosslinks (also known as protein adducts) to produce clean DNA ends necessary for proper repair. In higher eukaryotes, TDP2 is the sole enzyme that has been identified to possess the activity to remove 5’-tyrosine adducts from double-stranded oligonucleotide substrates [51, 52]. By using a specific oligonucleotide to study irreversible TOP2 cleavage complexes, Rui Gao and colleagues demonstrate that native TOP2 cleavage complexes first need to be proteolyzed before they can be processed by TDP2 [53]. In this investigation, we discovered that MDM2 inactivation increased the levels of stable TOP2βcc and triggered a time-dependent accumulation of TOP2βcc, strongly indicating the role of MDM2 in the proteolysis of native TOP2β cleavage complexes (Fig. 4). Indeed, inactivation of MDM2 ubiquitin ligase blocks TOP2β degradation to mask DSBs and subsequently inhibits the DNA damage response (Fig. 6). Consistently, cancer cell lines that overexpress MDM2 show attenuated levels of γH2AX induced by TOP2 poisons [11]. Recently, we discovered that teniposide targets the DNA damage signal and actives ATM, collaborating with CK1 to phosphorylate TOP2β on Ser1134 and Ser1130 to facilitate β-TrCP binding and subsequent degradation [10, 54]. Thus, it is conceivable that MDM2 and β-TrCP appear to cooperatively interact with TOP2β to accelerate the proteolysis of the native TOP2β cleavage complex, which allows TDP2 to remove 5’-tyrosine adducts and creates 5’-phosphate termini that can be used directly for re-ligation.

It is generally acknowledged that DNA damage triggers fast phosphorylation-mediated MDM2 degradation, leading to a temporary decrease in MDM2 levels, allowing p53, a guardian of the genome, to accumulate and become fully activated to maintain genomic stability [55, 56]. Furthermore, SCF^β-TrCP^ was identified as the E3 ubiquitin ligase is involved in ubiquitinating and destroying MDM2 [57]. In addition, MDM2 also regulates the DNA DSBs repair response and genome integrity independently of p53 by the ubiquitination of the HBP1 transcription factor and interacting with Nbs1, a component of the Mre11/Rad50/Nbs1 DNA DSB repair complex [24, 25]. Additionally, our study identified a new mechanism by which MDM2 regulates TOP2 poison-induced genome stability by ubiquitinating TOP2β. However, inconsistently, we found that upon VP-16 stimulation, the level of MDM2 expressed was increased considerably in the tested cells, especially in A549 cells, along with an increased protein level of p53 (Fig. 1A, B). Remarkably, the MDM2 gene is transcriptionally activated by p53 [27]. The continual production and destruction of the MDM2 protein in homeostasis may partially explain this finding. Upon DNA damage, a dual mechanism unfolds. Firstly, it instigates the degradation of the MDM2 protein. Simultaneously, this triggers the activation of wild-type p53, thereby orchestrating the transcription of the MDM2 gene. Consequently, this intricate interplay leads to the early induction of MDM2. Furthermore, by employing siRNA-mediated knockdown of p53 in A549 cells, we further confirmed that the levels of p53 do not impact the interaction between MDM2 and TOP2β, nor do they affect the degradation of the TOP2β (Figs. 1D, F and 2D, H). In addition, a slight upregulation of MDM2 mRNA and protein levels was also observed in p53-null H1299 cells, indicating that other unidentified transcription factors may be involved in the regulation of MDM2 on VP-16-induced DNA damage. On the other hand, it has been reported that DNA damage does not result in faster MDM2 degradation [58], and the spurious phenotype of MDM2 decay following DNA damage is attributed to phosphorylation-based epitope masking, resulting in SMP14 and 2A10, two phosphorylation-sensitive antibodies against MDM2 that identify strong MDM2 suppression following DNA damage [58]. Thus, the MDM2 antibody obtained from Calbiochem that is insensitive to phosphorylation was used in the study. In addition, MDM2 has been found to trigger TOP2α‘s ubiquitination and degradation [5, 59]. In this study, we did not find a relationship between MDM2 and TOP2α degradation. This finding may be explained by TOP2α being ubiquitinated only by MDM2 in cells with a single nucleotide polymorphism (SNP) in the MDM2 promoter that caused MDM2 to be upregulated.

It is worth noting that compared with other DNA-damaging agents, cancer cells exhibiting MDM2 overexpression are preferentially resistant to TOP2 poisons, and have decreased DNA DSBs in response to etoposide [11]. Furthermore, MDM2 levels as well as an active ubiquitin ligase are required for the selective decrease in DNA damage induced by these agents, but not p53 or TOP2α levels [11]. It was found by attaching to the 3’-UTR of MDM2, microRNA miR-181b consistently causes MDM2 to express less, making malignant cells more sensitive to teniposide, resulting in its decreased expression [12]. Nonetheless, uncertainty exists over underlying molecular mechanisms. In this work, we offer a reasonable theory that independent of p53 and TOP2α, MDM2 mediates the exposure of TOP2β-concealed DNA DSBs, initiating the DDR and DNA repair and enhancing survival by inducing the ubiquitination and subsequent degradation of TOP2β.

Both TOP2α and TOP2β are similarly suppressed by TOP2 poisons due to their identical catalytic mechanisms. However, TOP2α is the more abundant isoform that causes the cytotoxic effects of TOP2 poison in proliferating cells [6]. In contrast, TOP2β performs a key function in cardiotoxicity and cancer development caused by TOP2 poison because its degradation mediates TOP2 poison-induced DSBs [9, 60]. Notably, the cytotoxic TOP2cc levels in cancer cells may also drop as a result of TOP2β degradation, which can then result in the establishment of treatment resistance. Consistent with this notion, investigations have demonstrated that TOP2 poisons and proteasome inhibitors together significantly enhance their ability to kill cells, showing that suppressing TOP2α and TOP2β degradation may improve the effectiveness of TOP2 poisons [61]. Nonetheless, the combination of TOP2 poisons and proteasome inhibitors is likely unsafe due to the significant toxicity to normal cells. Because of this, selective suppression of E3 ligases that control TOP2 stability might probably be preferable to systemic ubiquitin-proteasome system (UPS) inhibition, leading to increased therapeutic effectiveness and fewer adverse effects. However, there are currently no effective small molecule inhibitors known to target SCF^β-TrCP^. In this study, we demonstrate that RG7112, a strong and specific MDM2 antagonist of the nutlin family blocks MDM2-mediated TOP2β degradation, in addition to MDM2, and may also regulate the stabilization of TOP2α. Furthermore, we demonstrate the combination activity of VP-16 and RG7112, in the xenograft tumor model and the in-situ lung cancer mouse model. The combinatorial regimens (RG7112 + VP-16) therapy significantly reduced the relative tumor volume and the relative tumor weight and retarded cell proliferation, induced more extensive intra-tumoral apoptosis in xenograft tumor model and in situ lung cancer mouse model (Fig. 8). Collectively, our research identifies a novel TOP2 poison treatment in combination with MDM2 inhibitors that impede TOP2β degradation for maximum malignant cell killing efficiency.

**Fig. 9:**
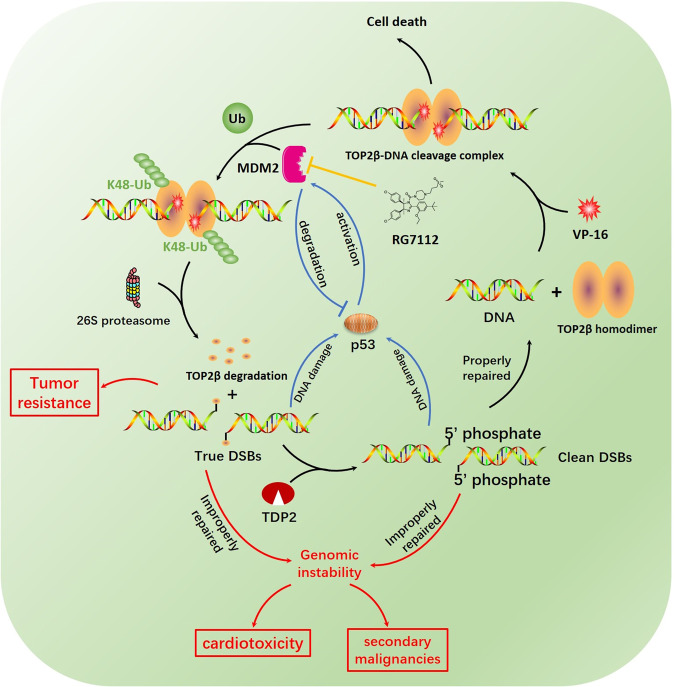
Working model. A working model for MDM2 provides TOP2 poison resistance by promoting proteolysis of TOP2βcc in a p53-independent manner.

In conclusion, our findings support a working model in which MDM2 mediates TOP2β degradation and eliminates TOP2β-DNA complexes following exposure to TOP2-concealed DNA DSBs, hence initiating the DDR and DNA repair and resulting in increased survival (Fig. 9). Specifically, once exposed to TOP2 poison, TOP2β binds to DNA to form TOP2βcc, thereby promoting MDM2 binding and subsequent TOP2β ubiquitination and degradation via the 26 S proteasome pathway. Biologically, by stabilizing TOP2βcc, MDM2 inactivation makes cells more sensitive to etoposide. Therefore, deactivating MDM2 in malignant cells, particularly those with wild-type p53, may increase the potency of TOP2 poisons, leading to fewer adverse effects and improved patient survival (Fig. 9).

### Supplementary information

Figiure s1

Supplementary legends

Original Data File

aj-checklist

## Data Availability

Original western blots are provided in Supplementary Material. The authors declare that all data supporting the findings of this study are available with the article or from the corresponding author upon reasonable request.
