# Correction: MDM2 provides TOP2 poison resistance by promoting proteolysis of TOP2βcc in a p53-independent manner

**DOI:** 10.1038/s41419-024-06634-5

**Published:** 2024-05-07

**Authors:** Jianfeng Shu, Jinni Jiang, Xiaofang Wang, Xuejie Yang, Guofang Zhao, Ting Cai

**Affiliations:** 1 Department of Thoracic Surgery, Ningbo No.2 Hospital, Ningbo, 315010 Zhejiang China; 2 Ningbo Institute of Life and Health Industry, University of Chinese Academy of Sciences, Ningbo, 315000 Zhejiang China

**Keywords:** Ubiquitin ligases, Chemotherapy, Proteases

Correction to: *Cell Death and Disease* 10.1038/s41419-024-06474-3, published online 23 January 2024

The original version of this article unfortunately contained a mistake. In the Original Data File, Fig. 1B CHK1 has been incorrectly labeled, and Fig. 5C ACTIN does not match precisely with the corresponding bands in the main manuscript. Moreover, the Original Data File comprises unpublished images pertaining to other subjects. These errors do not affect any of the findings reported in the paper. The final version of Original Data File are now provided. The authors apologize for any confusion that this error may have caused. The original article has been corrected.

Correct Fig. 1B
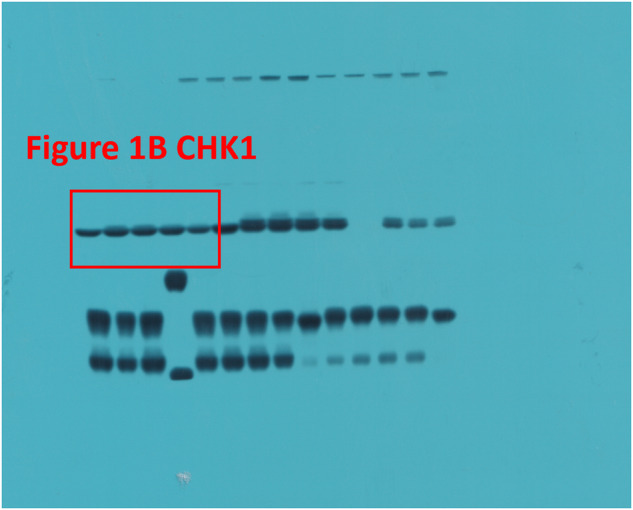


Incorrect Fig. 1B
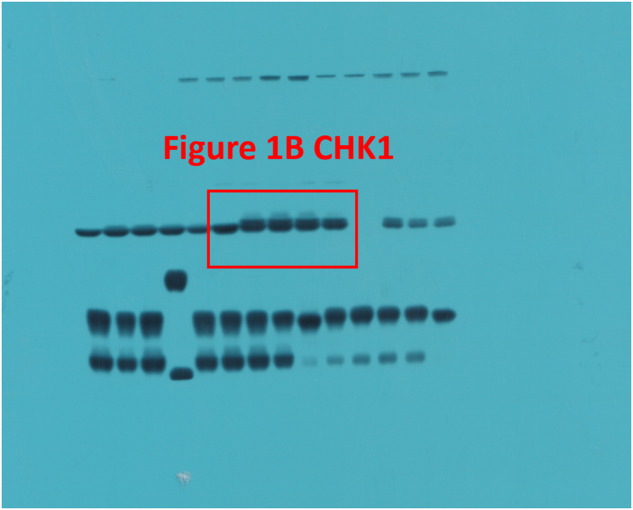


Correct Fig. 5C
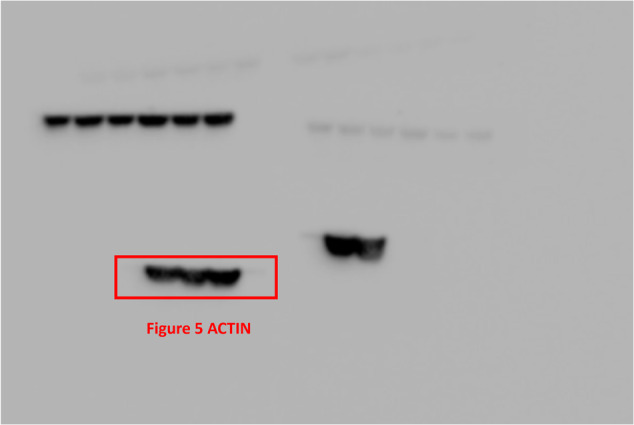


Incorrect Fig. 5C
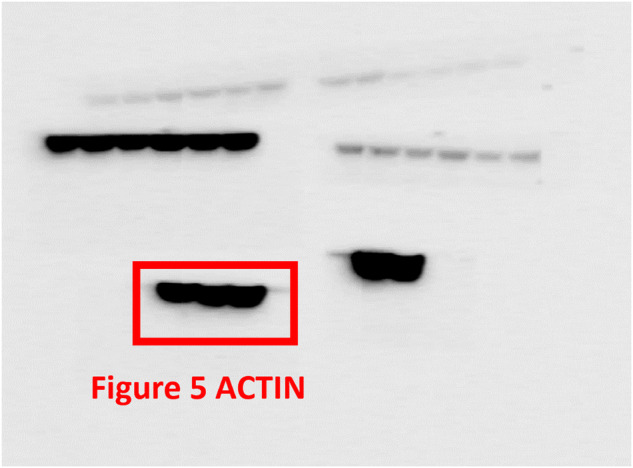


### Supplementary information

Supplementary Figures

