# Supplementary figures and images for: MDM2 provides TOP2 poison resistance by promoting proteolysis of TOP2βcc in a p53-independent manner
Source: Cell Death Dis. 2024 Jan 23;15(1):83. doi: 10.1038/s41419-024-06474-3 (PMC10806188; doi:10.1038/s41419-024-06474-3)

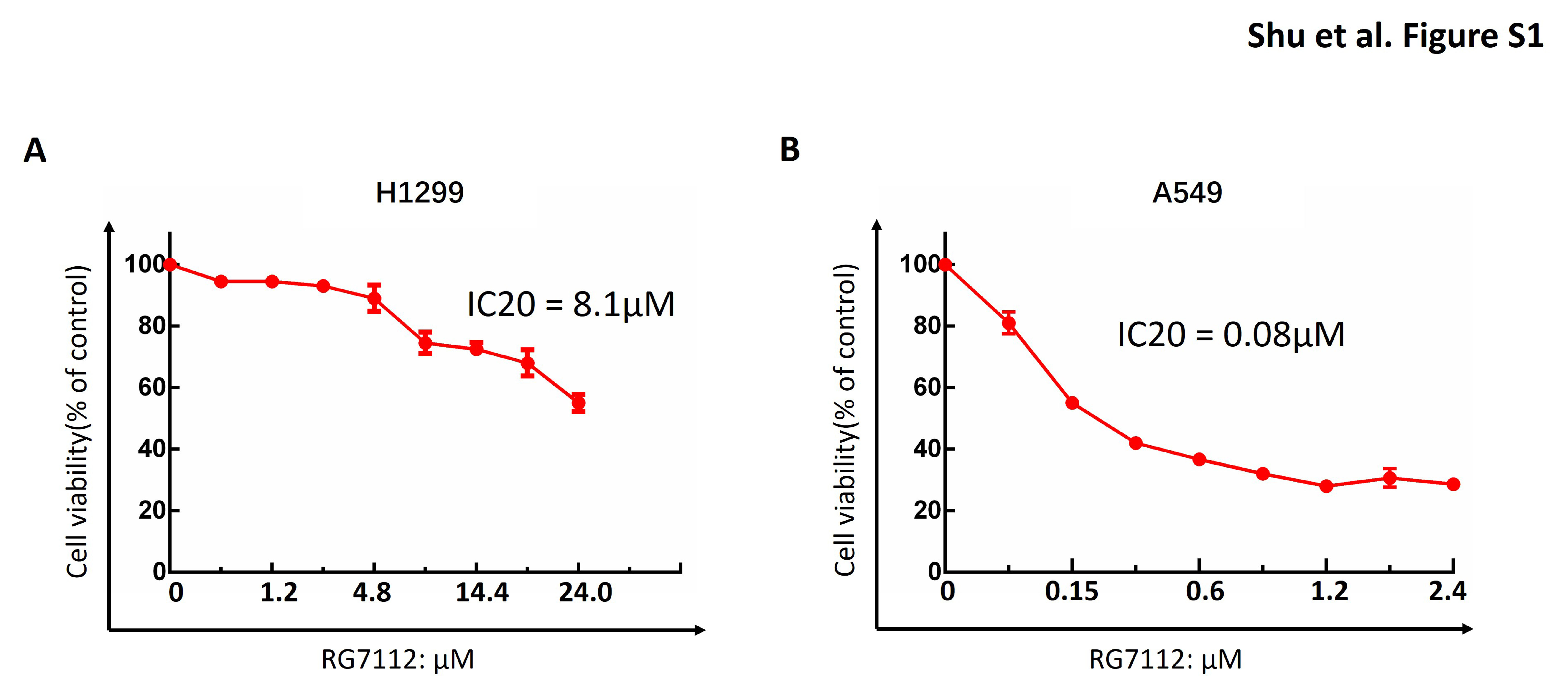

Supplement: Supplementary file 1 — Figiure s1 [file 41419_2024_6474_MOESM1_ESM.jpg]
