# Supplementary material for: MDM2 provides TOP2 poison resistance by promoting proteolysis of TOP2βcc in a p53-independent manner
Source: Cell Death Dis. 2024 Jan 23;15(1):83. doi: 10.1038/s41419-024-06474-3 (PMC10806188; doi:10.1038/s41419-024-06474-3)
Supplement: Supplementary file 2 — Supplementary legends [file 41419_2024_6474_MOESM2_ESM.docx]

**Figure S1. IC20 values of H1299 and A549 against RG7112.** (A, B) H1299 and A549 cells were treated with various concentrations of RG7112 for 72 h, followed by the ATP-lite assay.
