# Supplementary material for: Correction: MDM2 provides TOP2 poison resistance by promoting proteolysis of TOP2βcc in a p53-independent manner
Source: Cell Death Dis. 2024 May 7;15(5):320. doi: 10.1038/s41419-024-06634-5 (PMC11076279; doi:10.1038/s41419-024-06634-5)

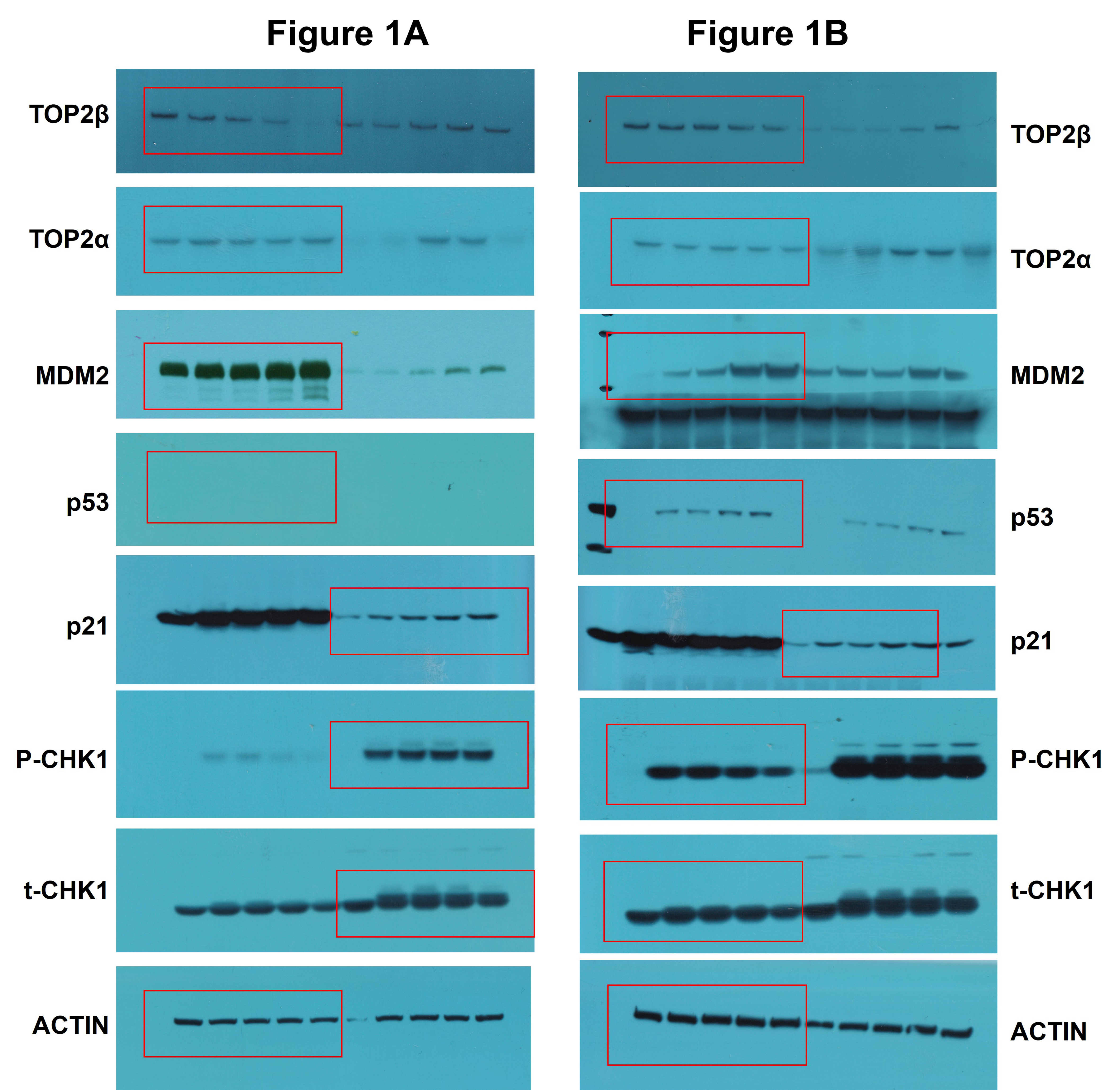
Figure 1A
Figure 1B
TOP2β
TOP2α
MDM2
p53
p21
P-CHK1
t-CHK1
ACTIN

Figure 1C

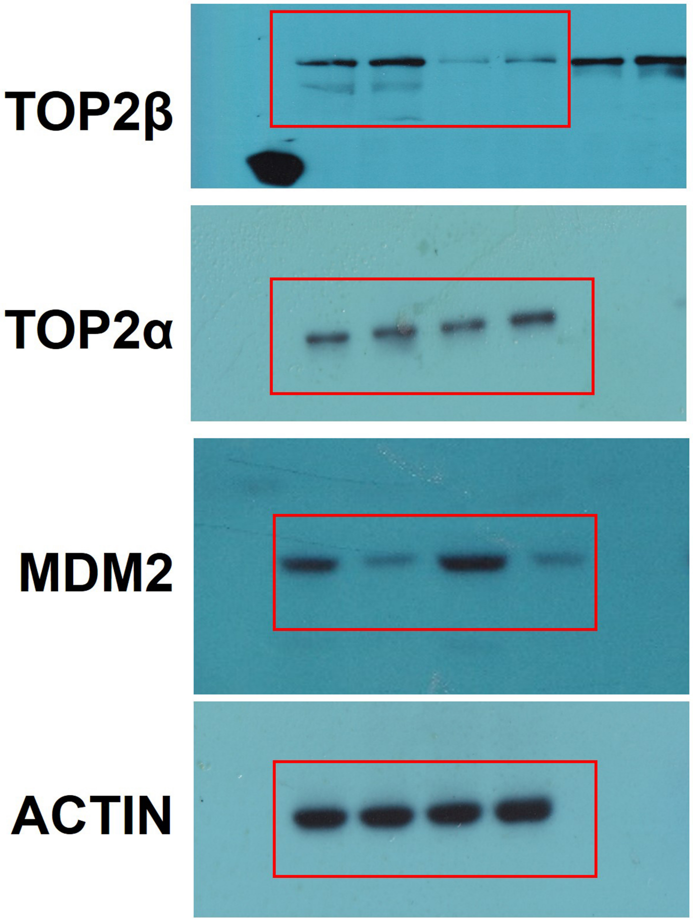

Figure 1D

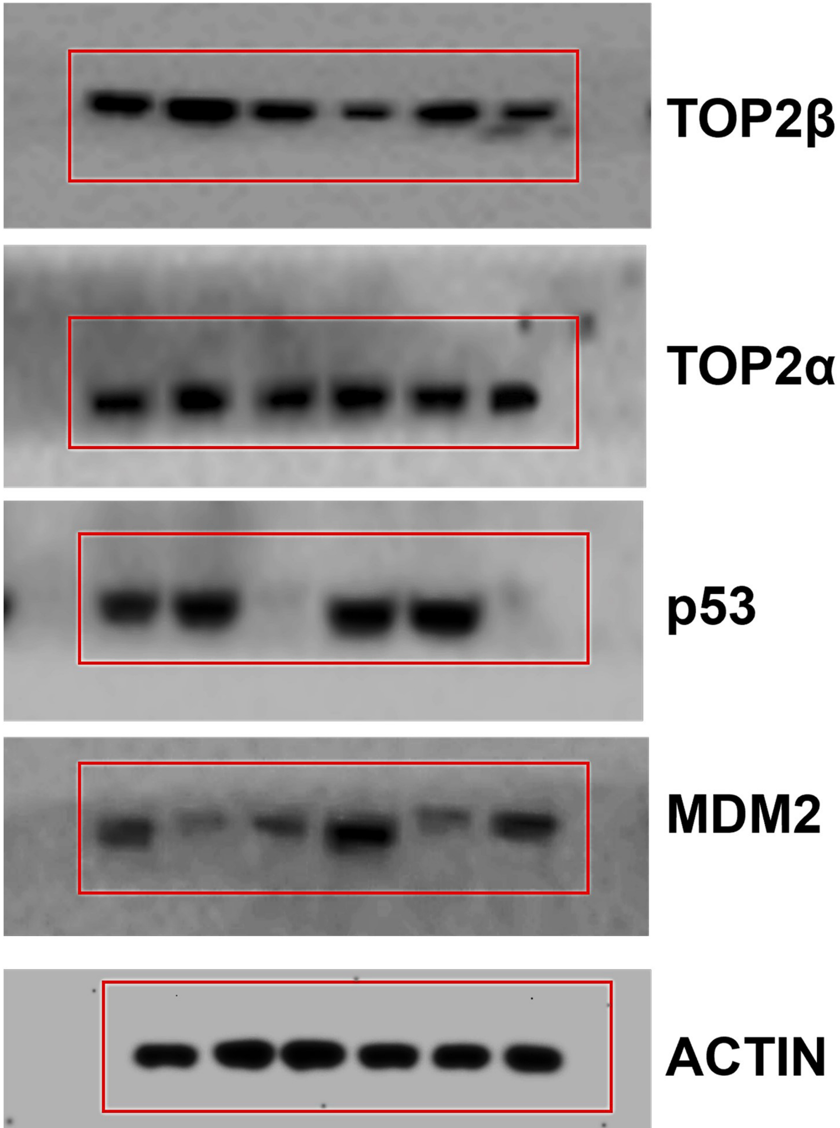

Figure 1E

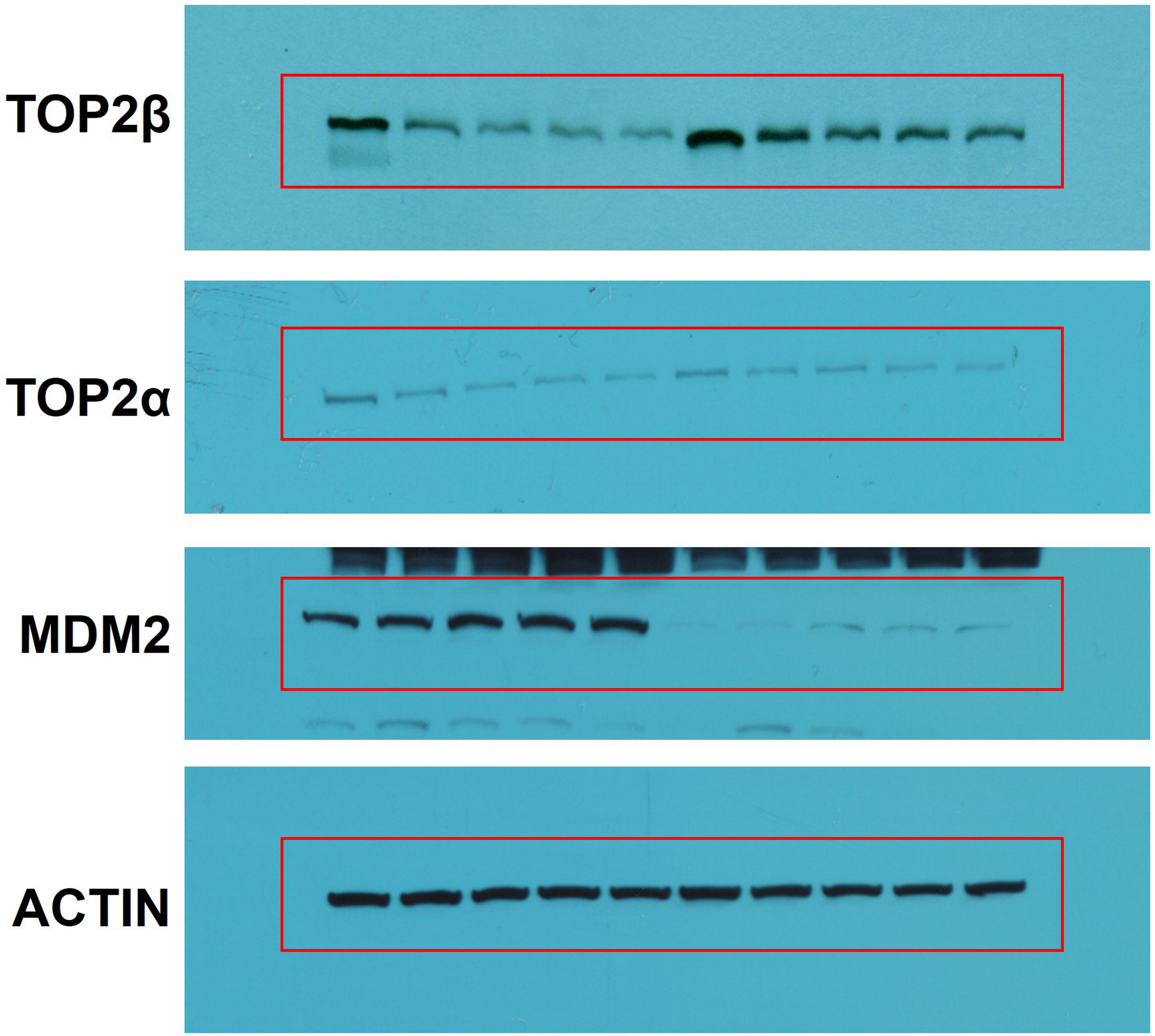

Figure 1F

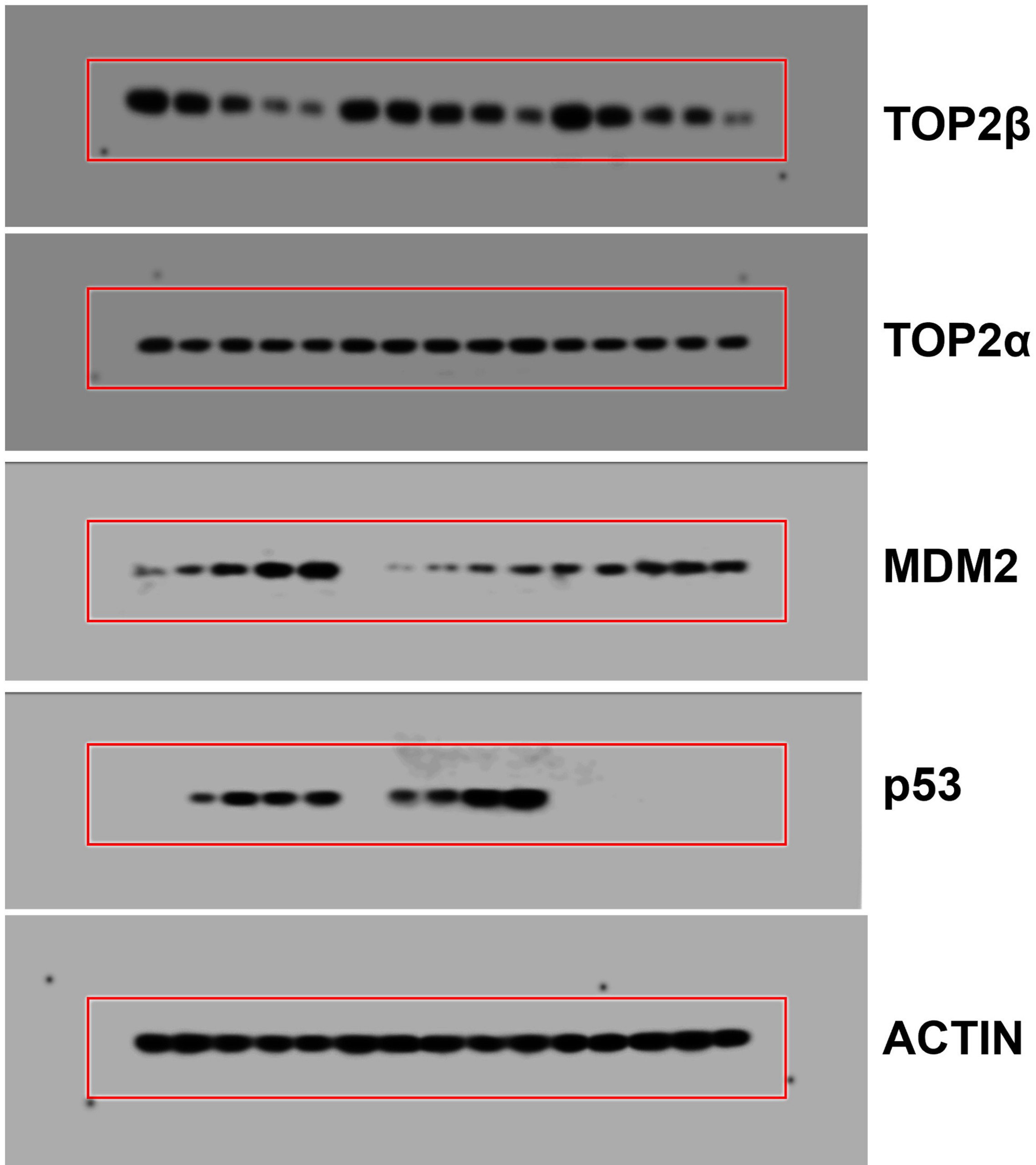

Figure 2A

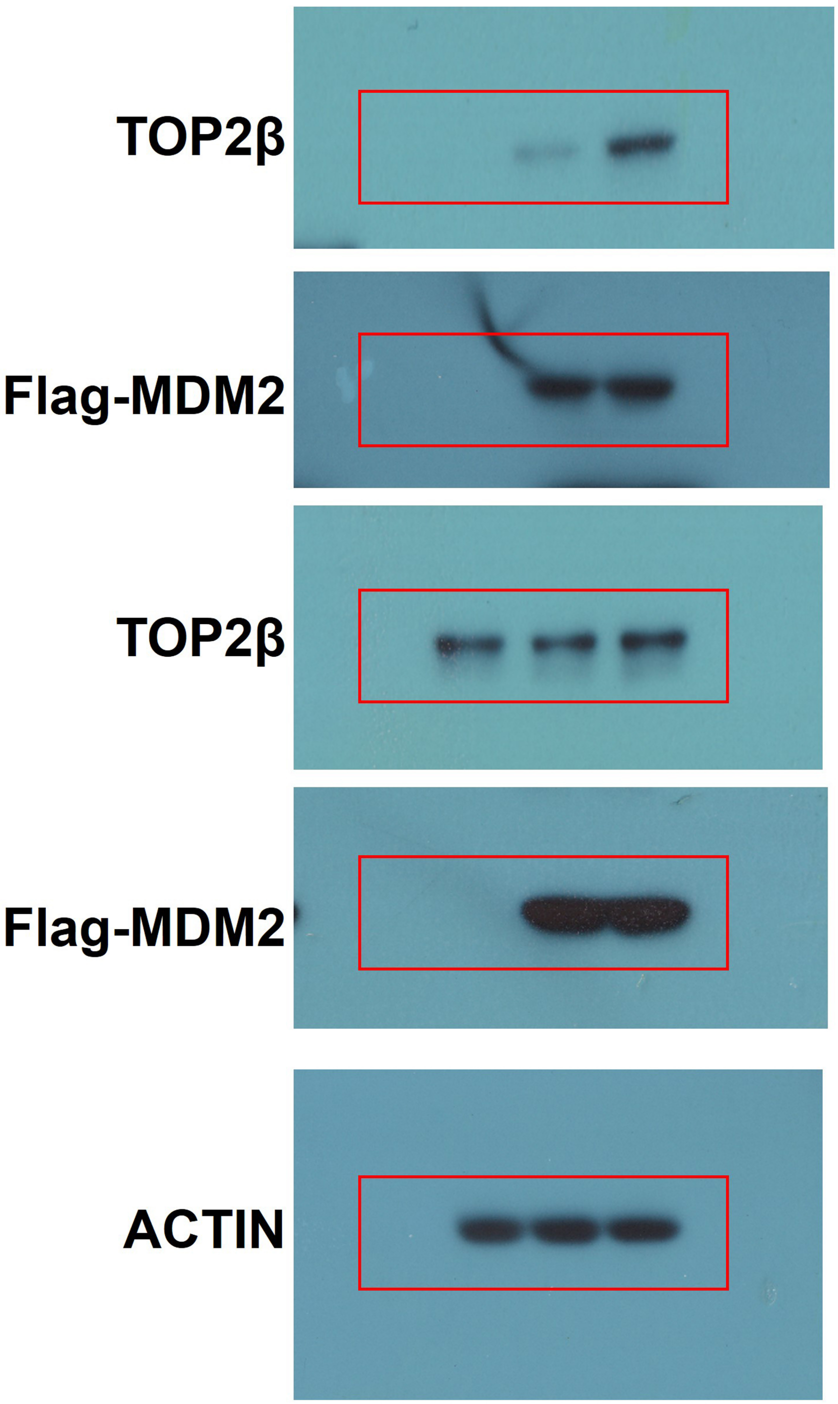

Figure 2B

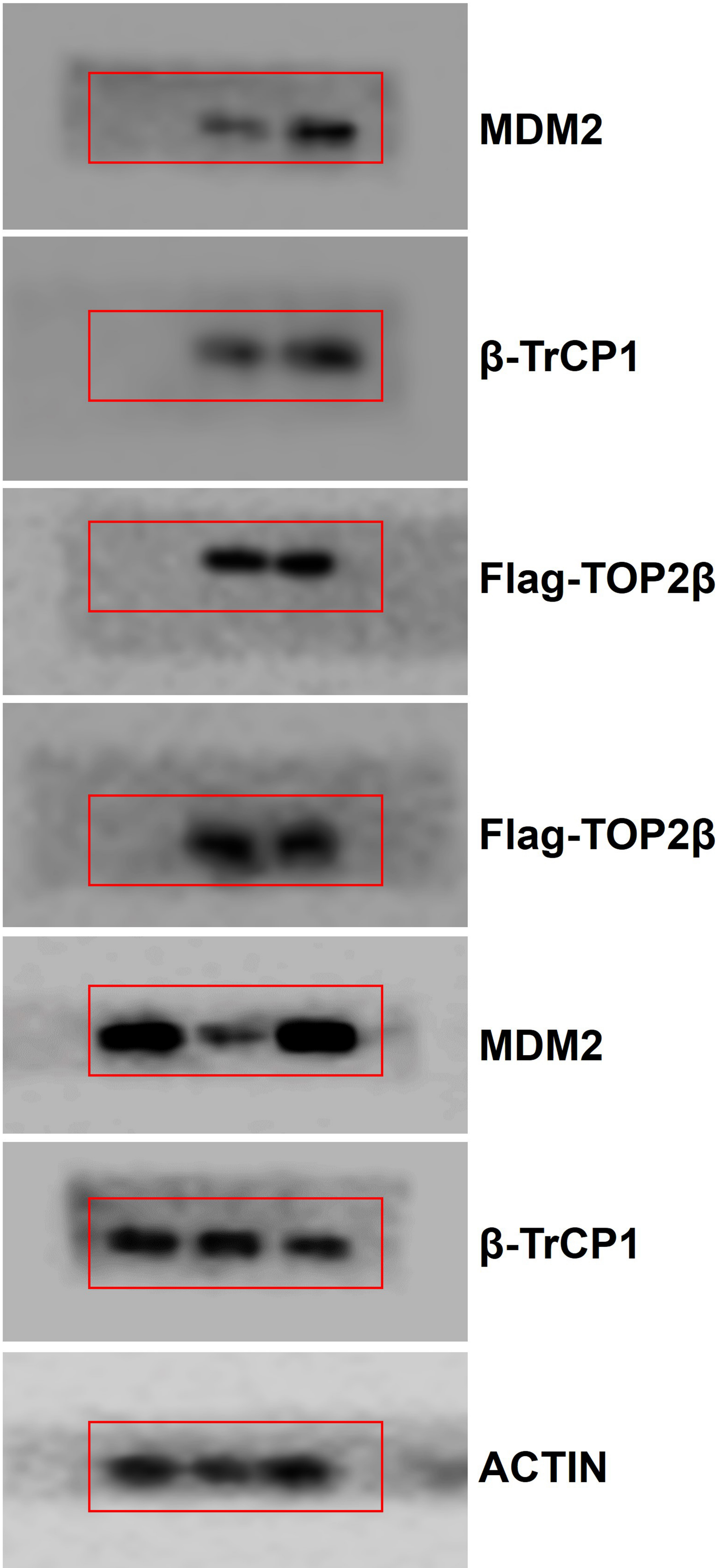

Figure 2C

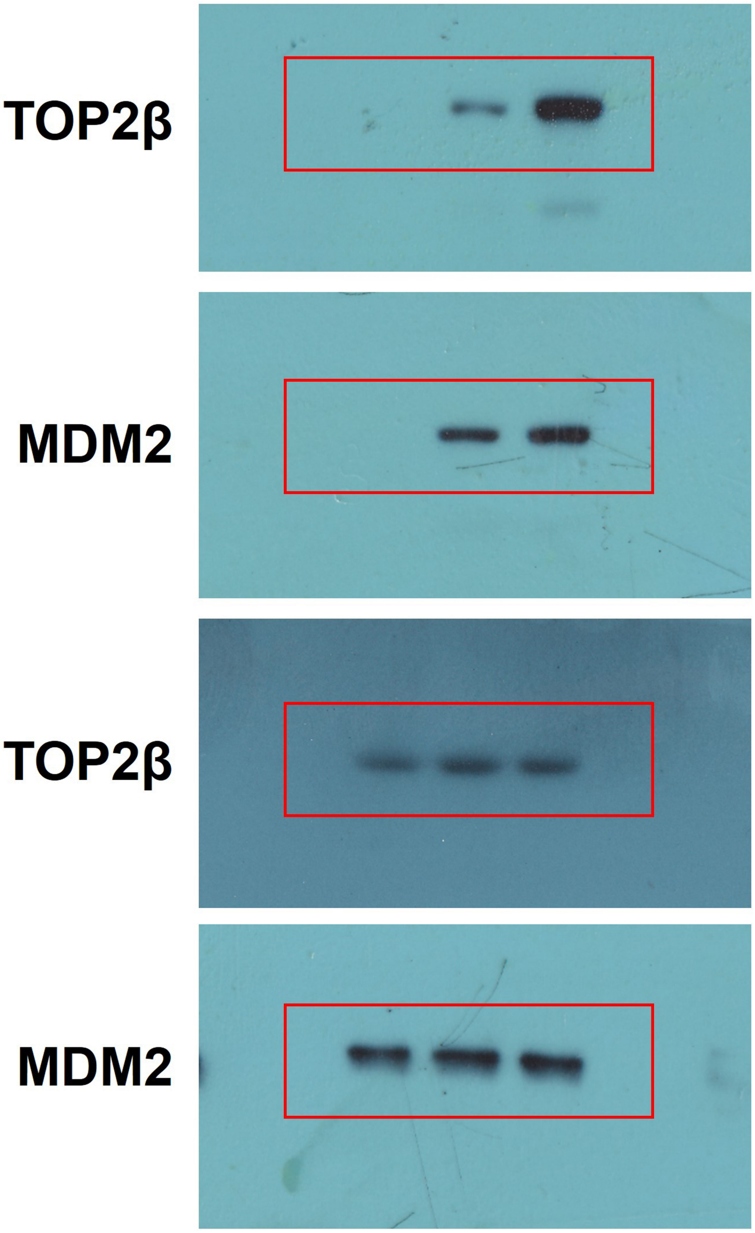

Figure 2D

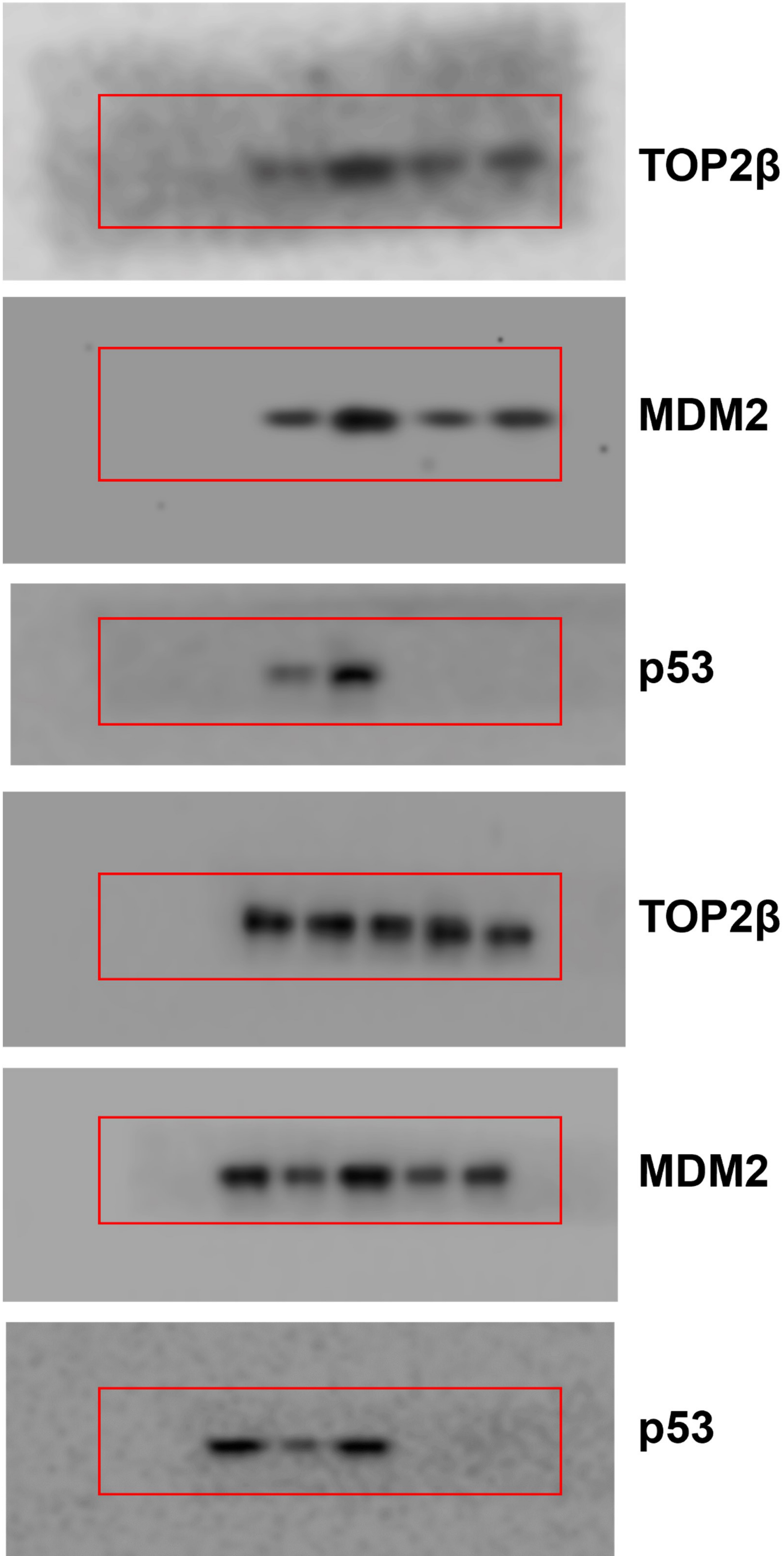

Figure 2G

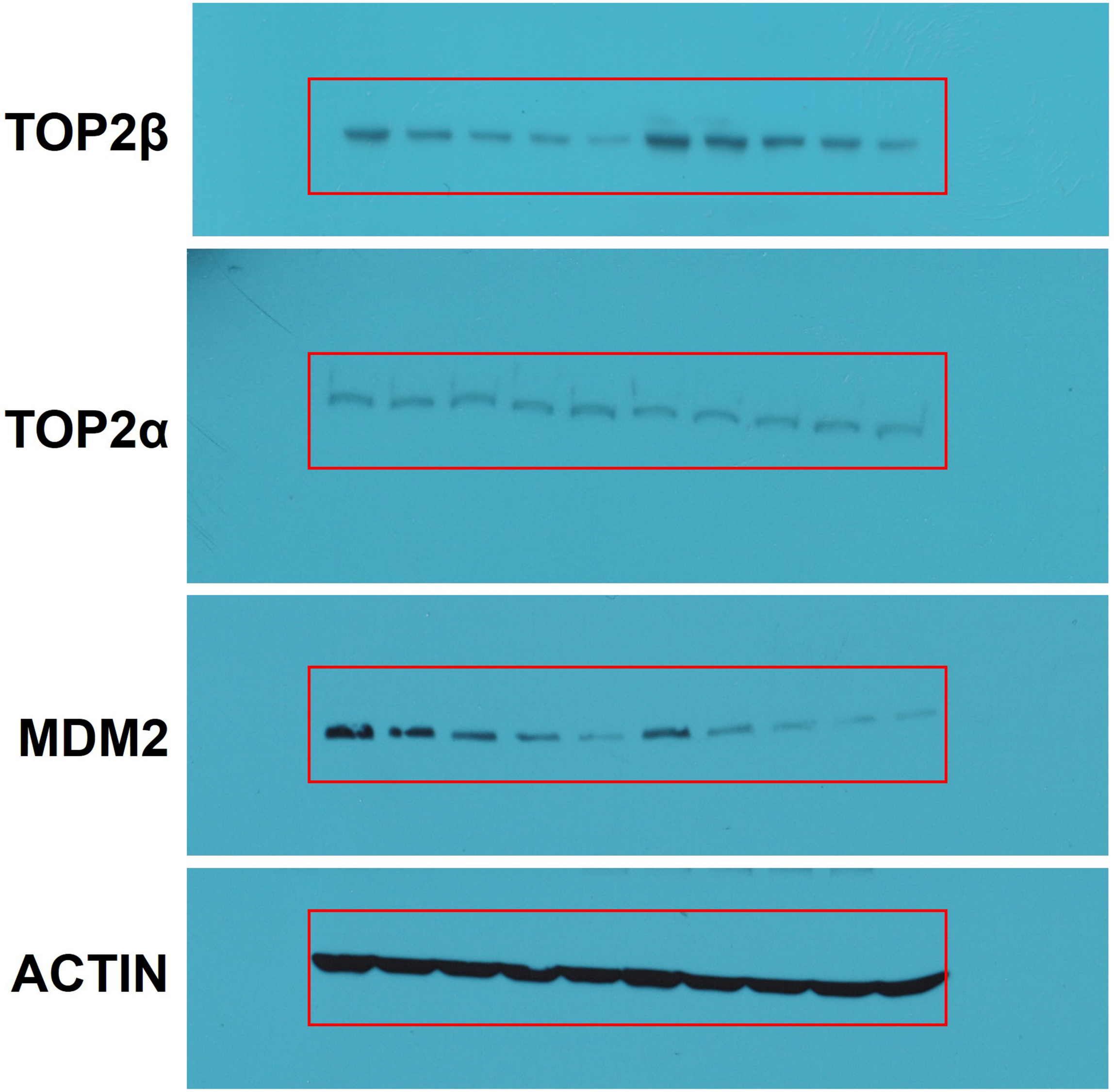

Figure 2H

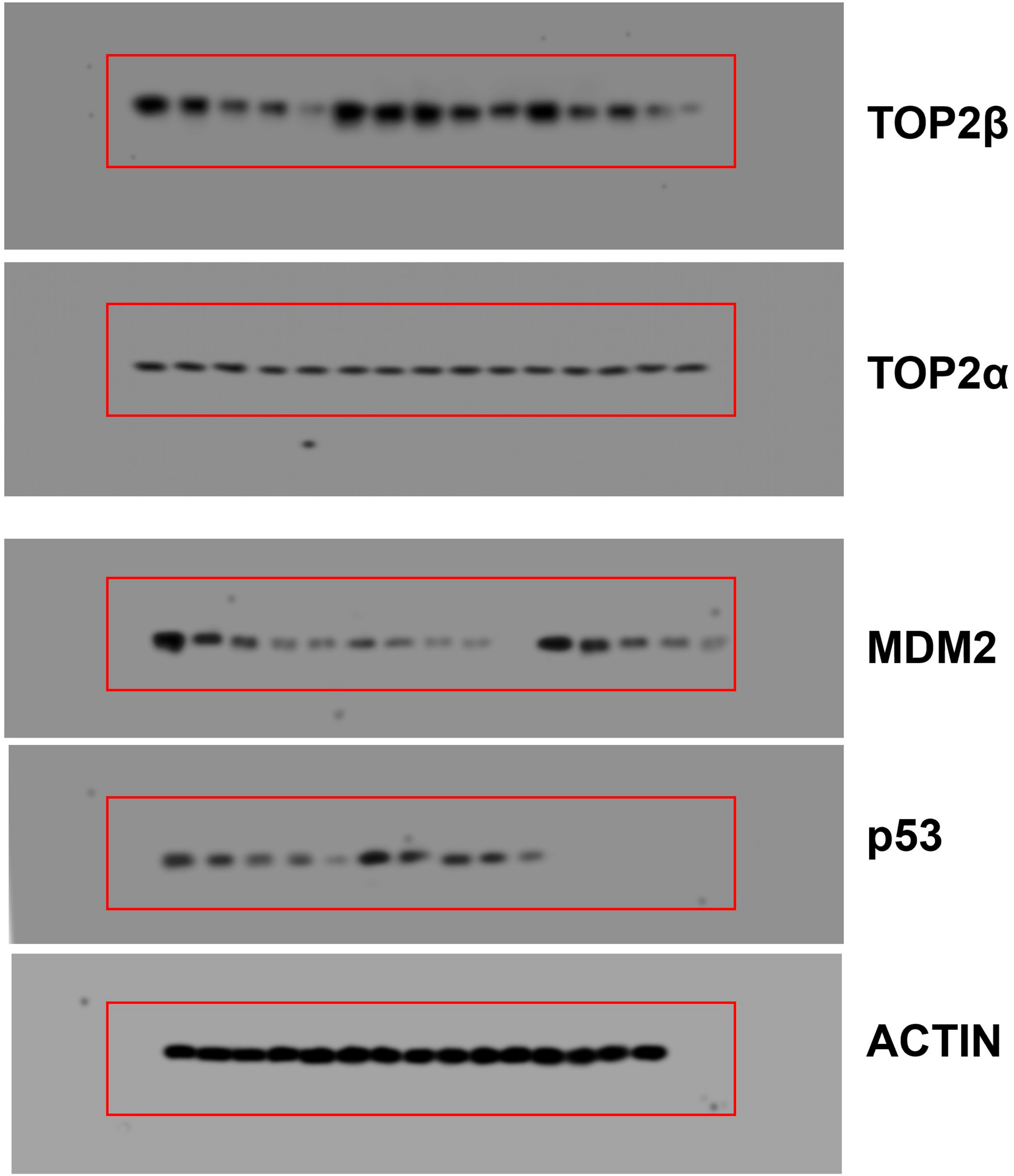

Figure 2I

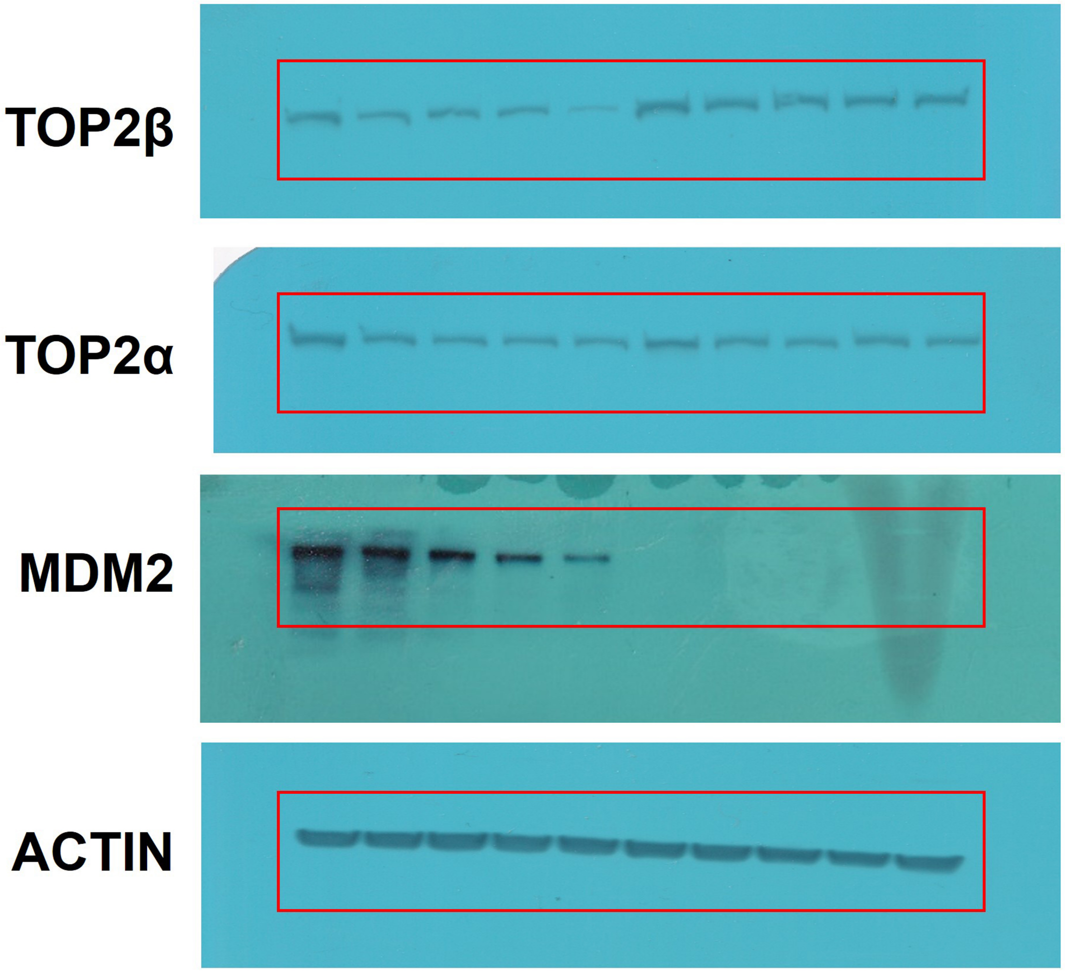

Figure 2I

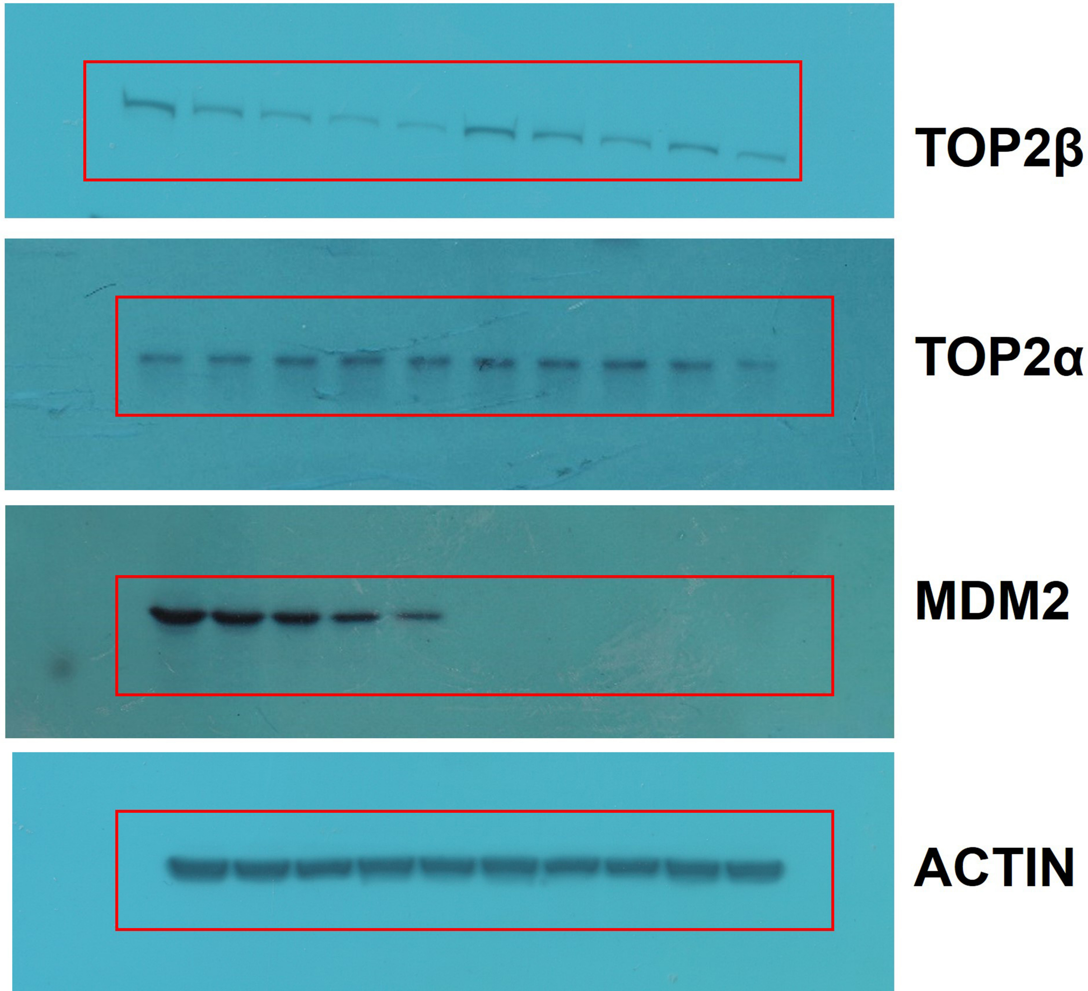

Figure 2J

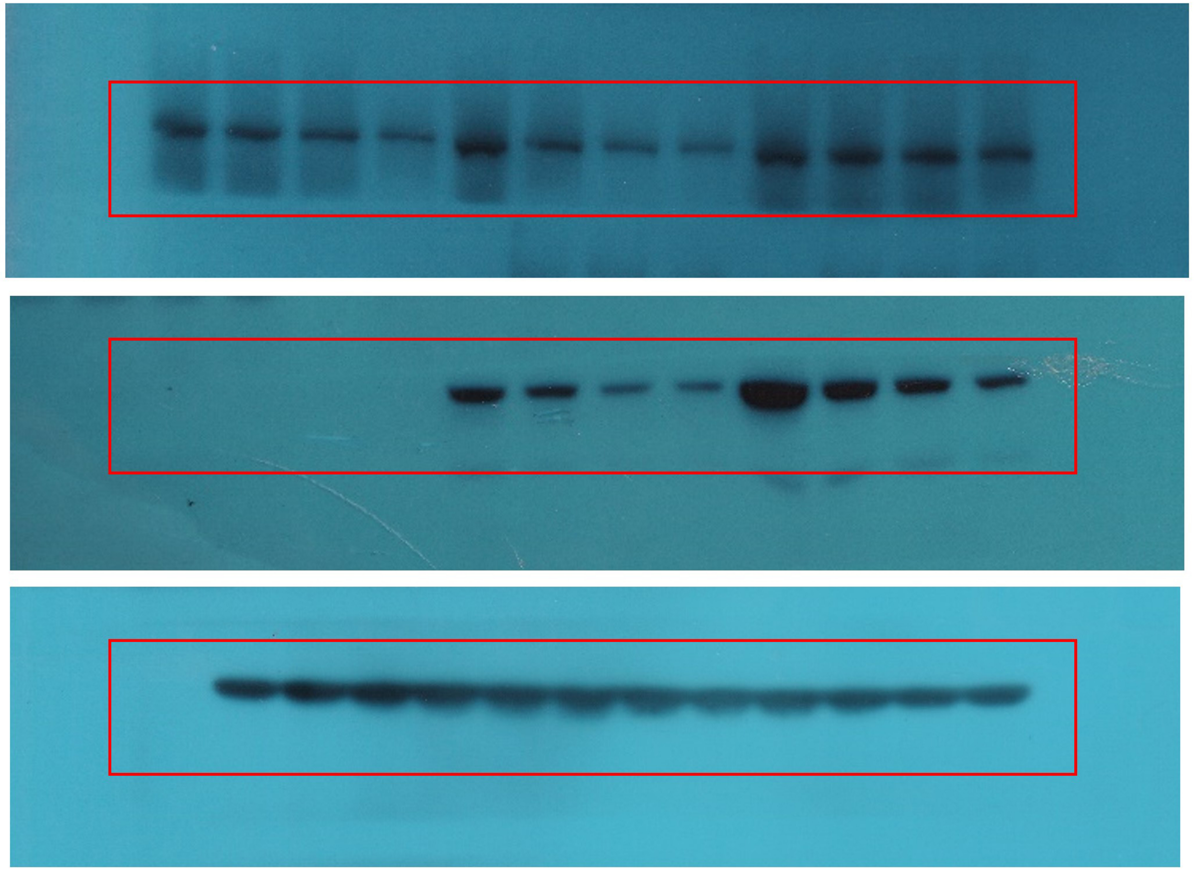

Figure 2K

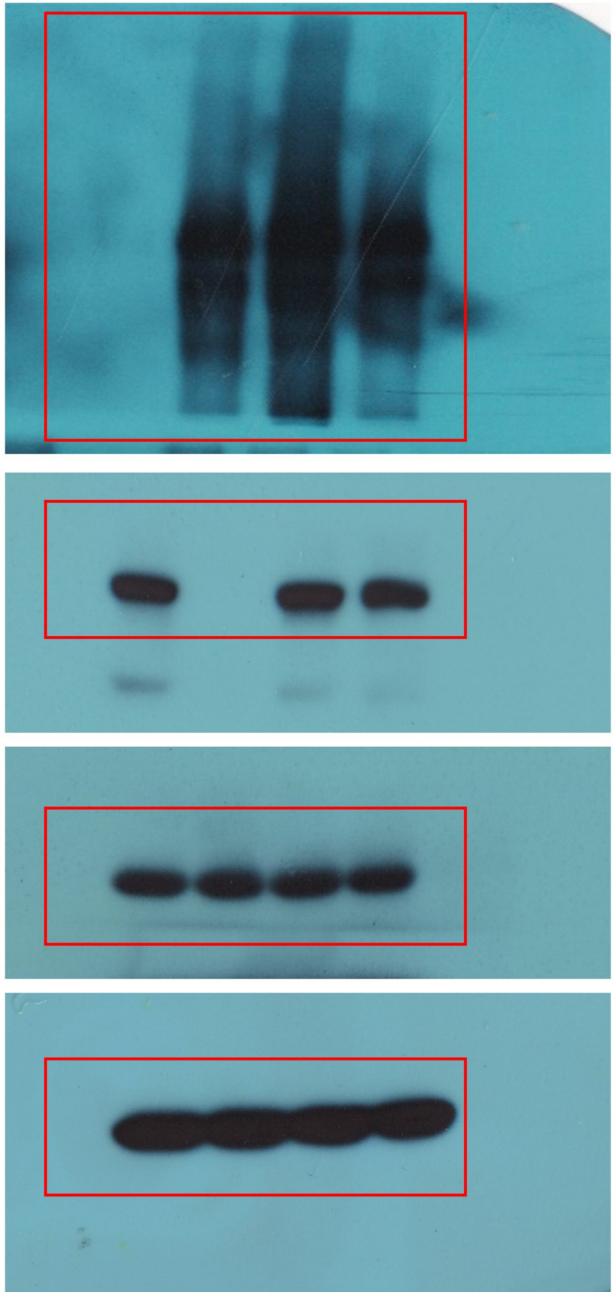

Figure 3A

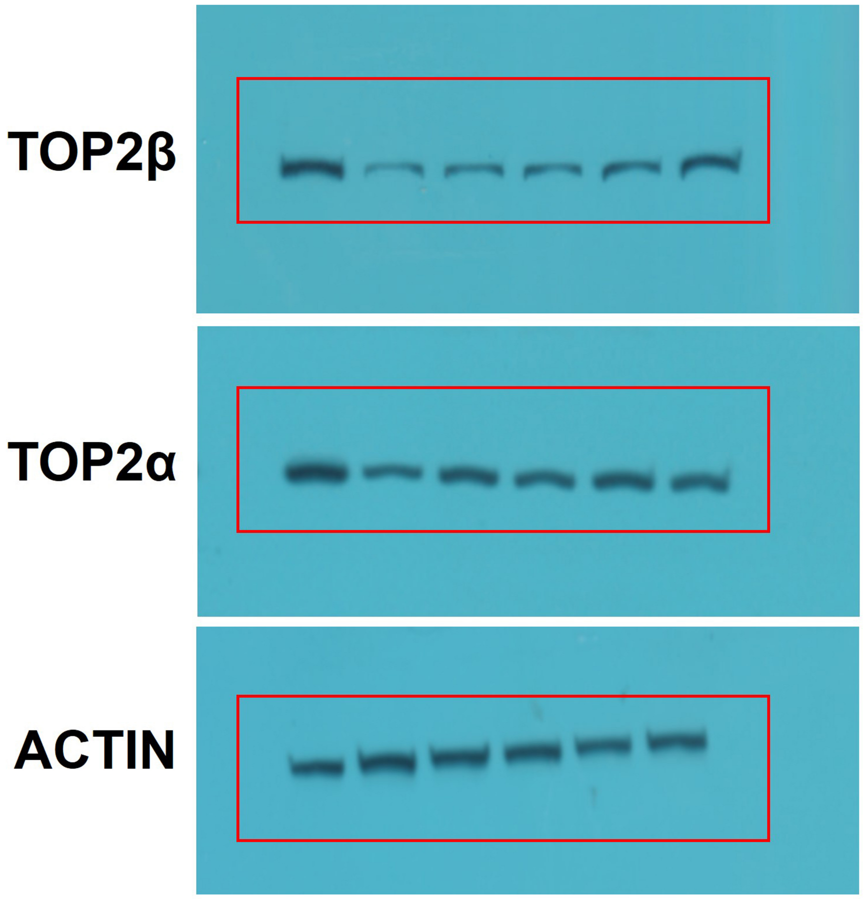

Figure 3C

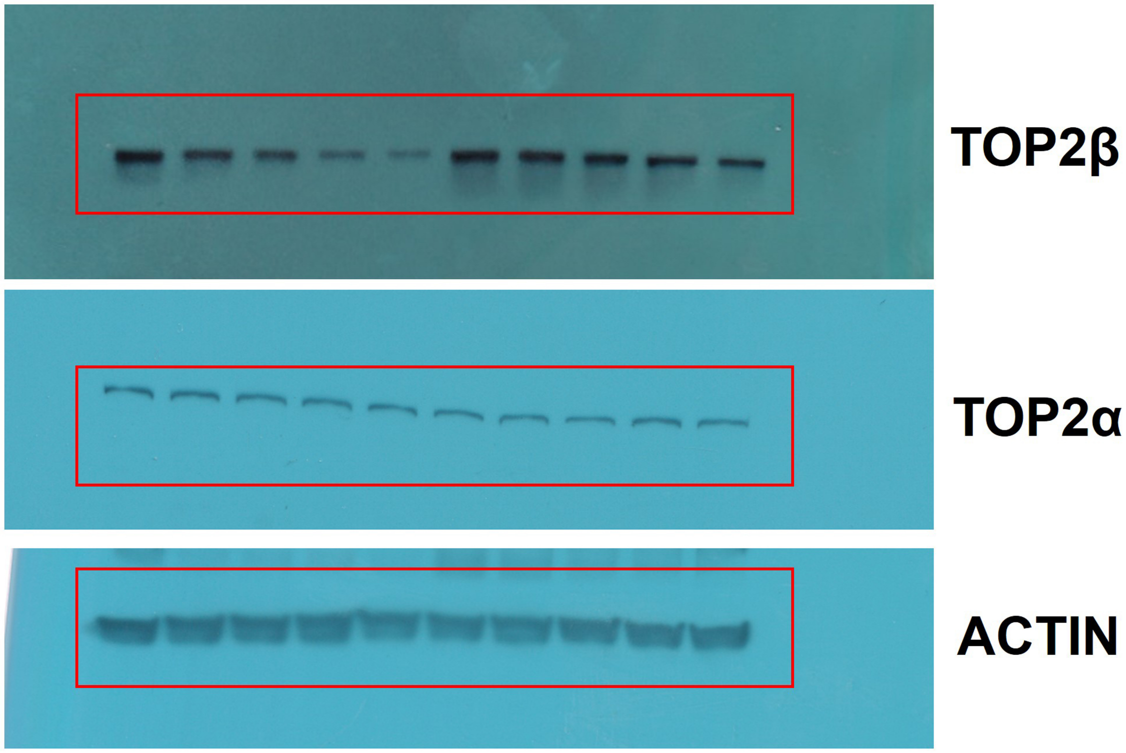

Figure 3B

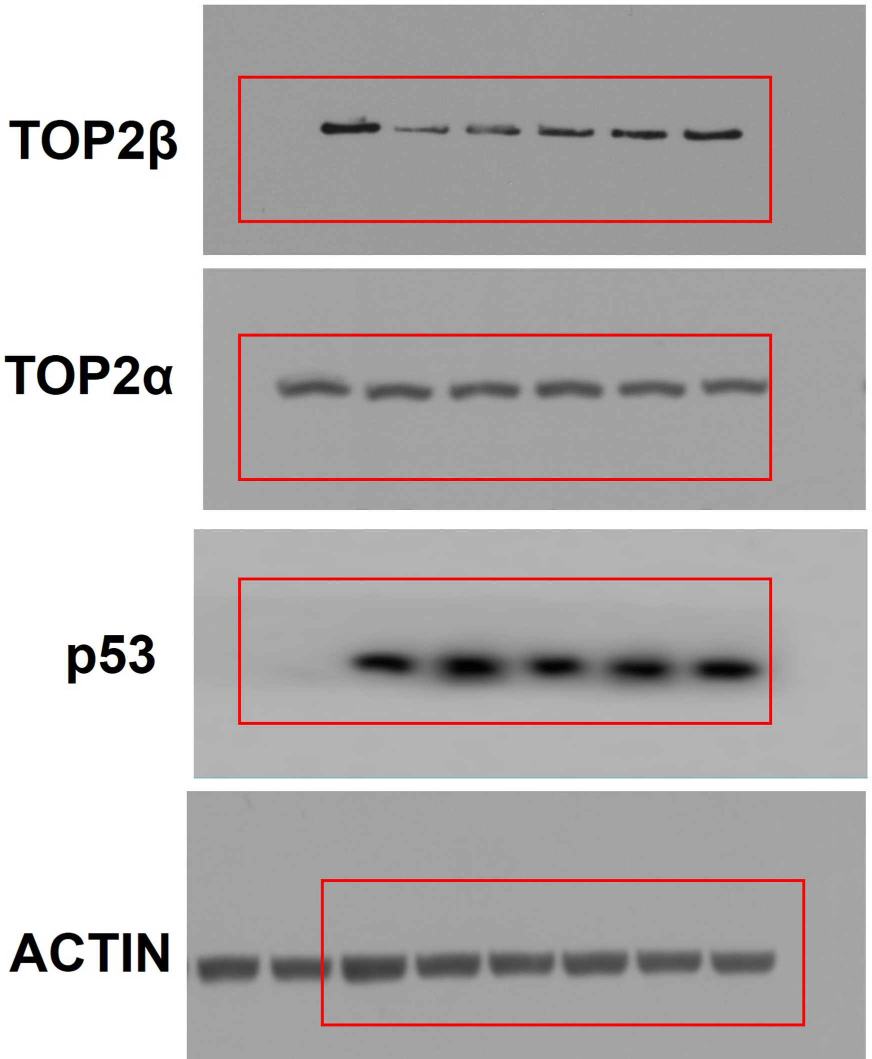

Figure 3D

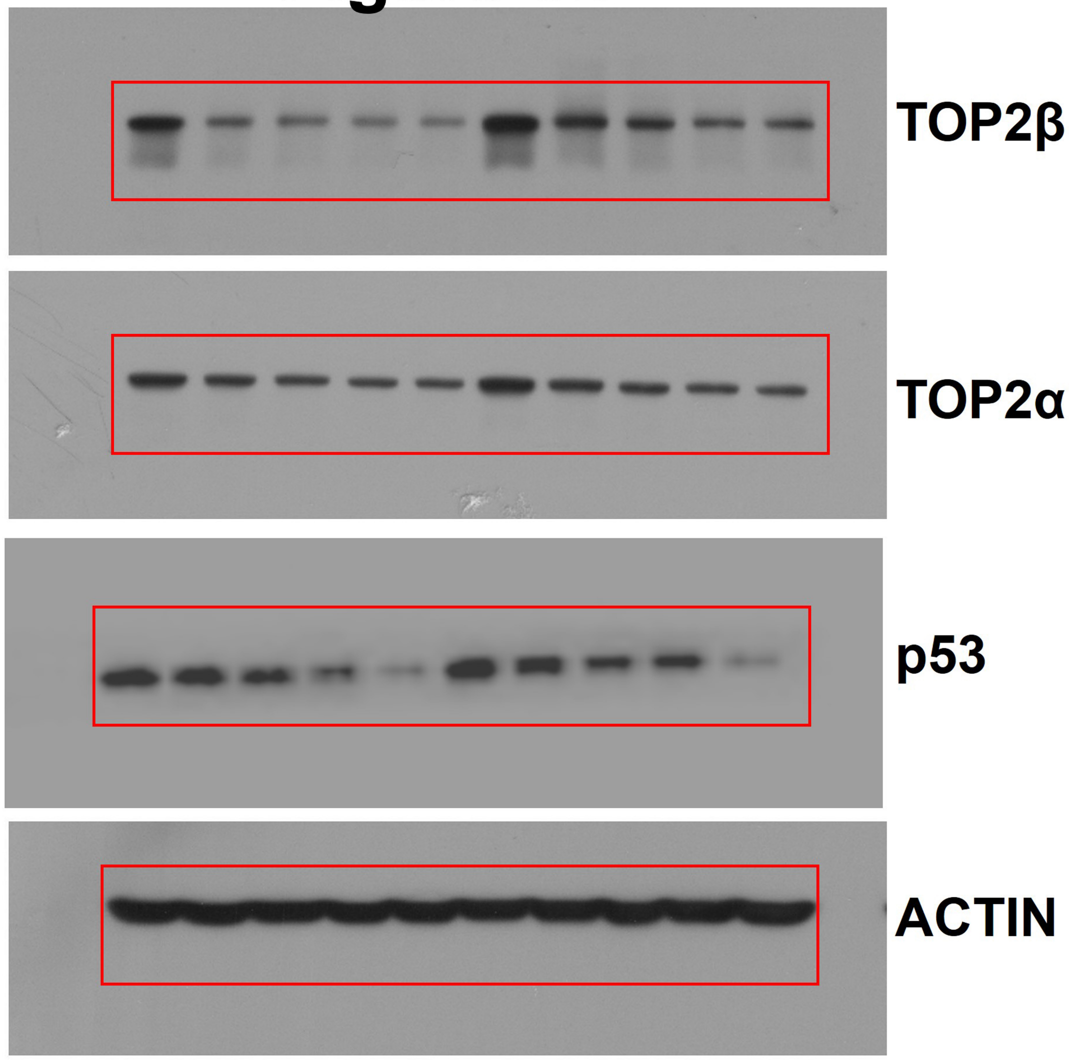

Figure 3E

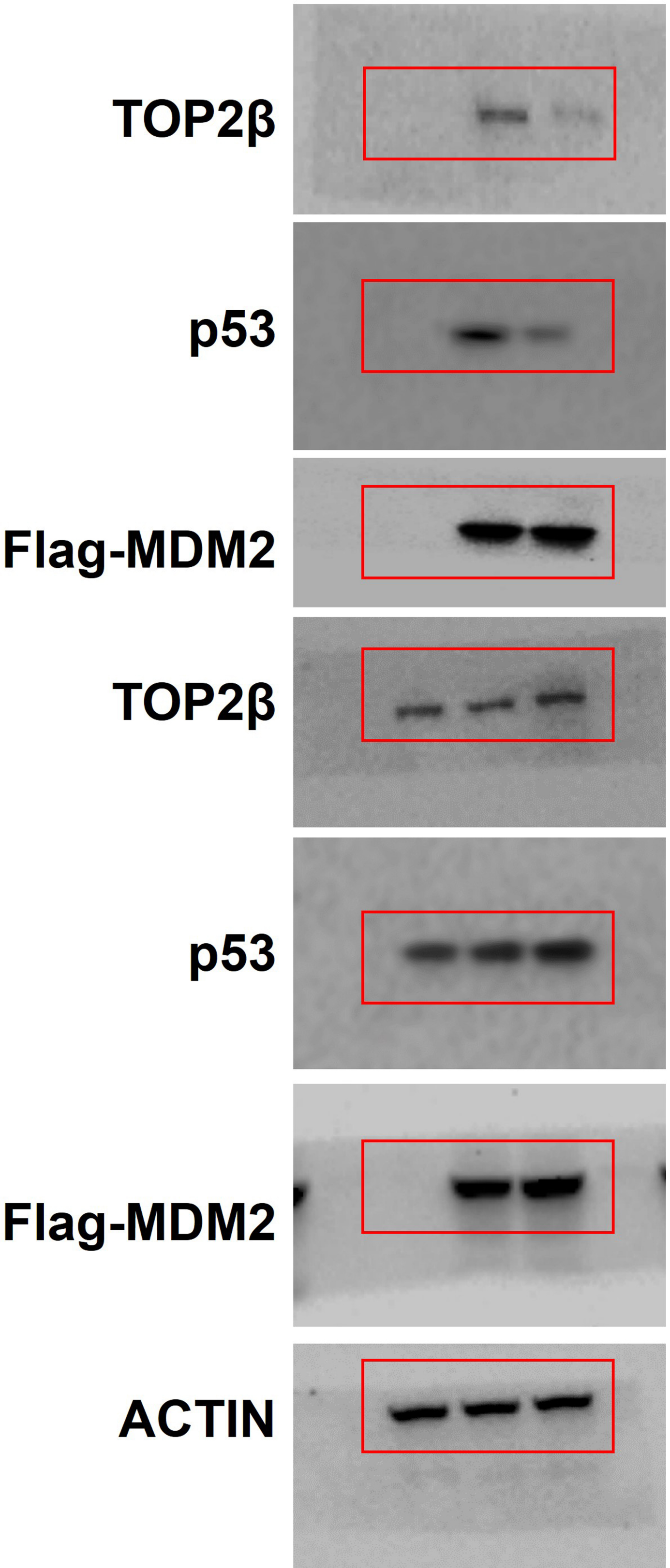

Figure 3F

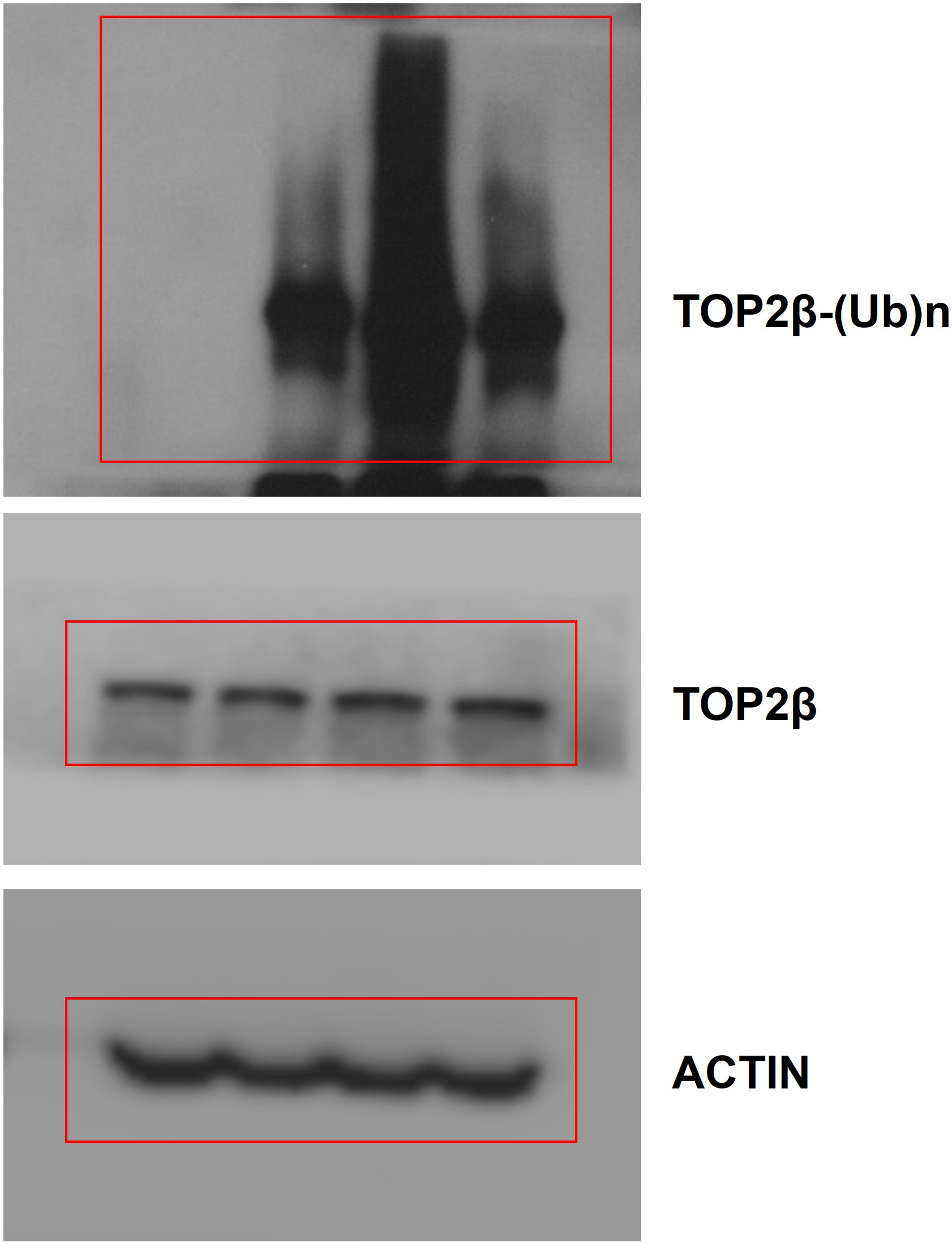

Figure 5A

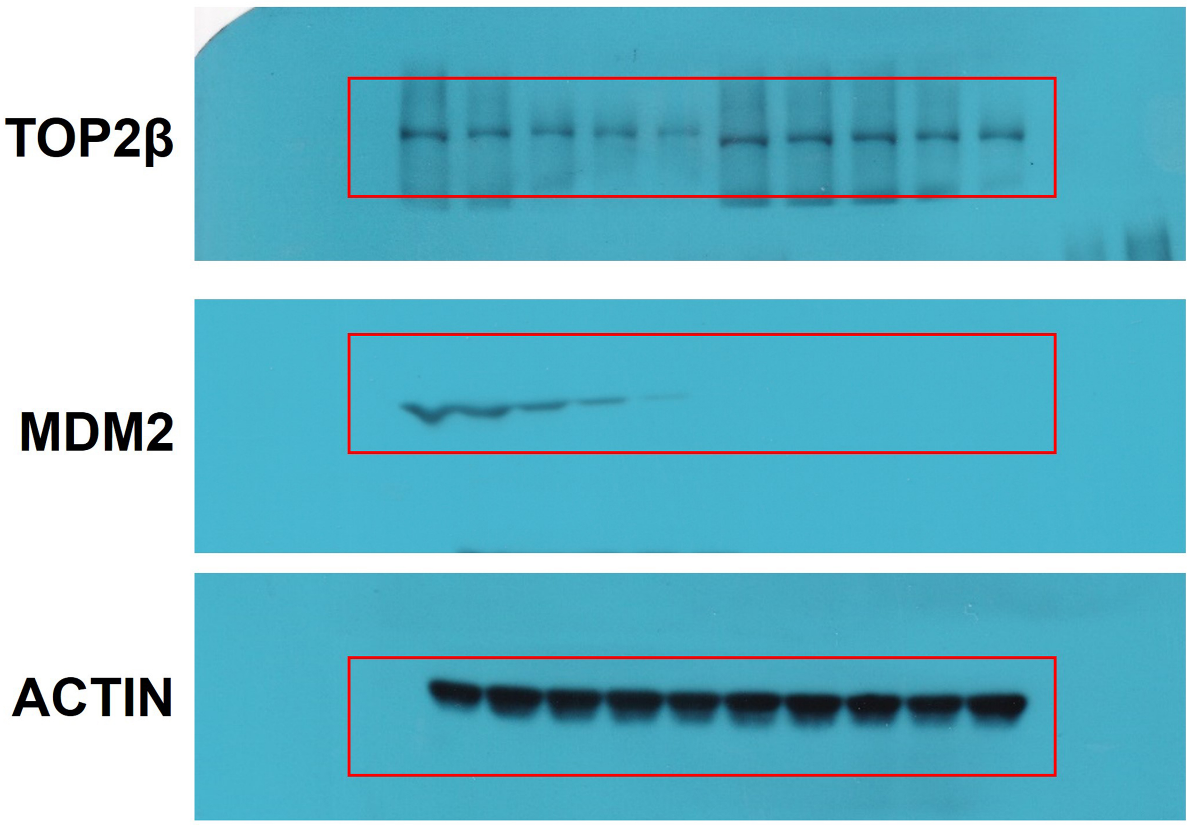

Figure 5B

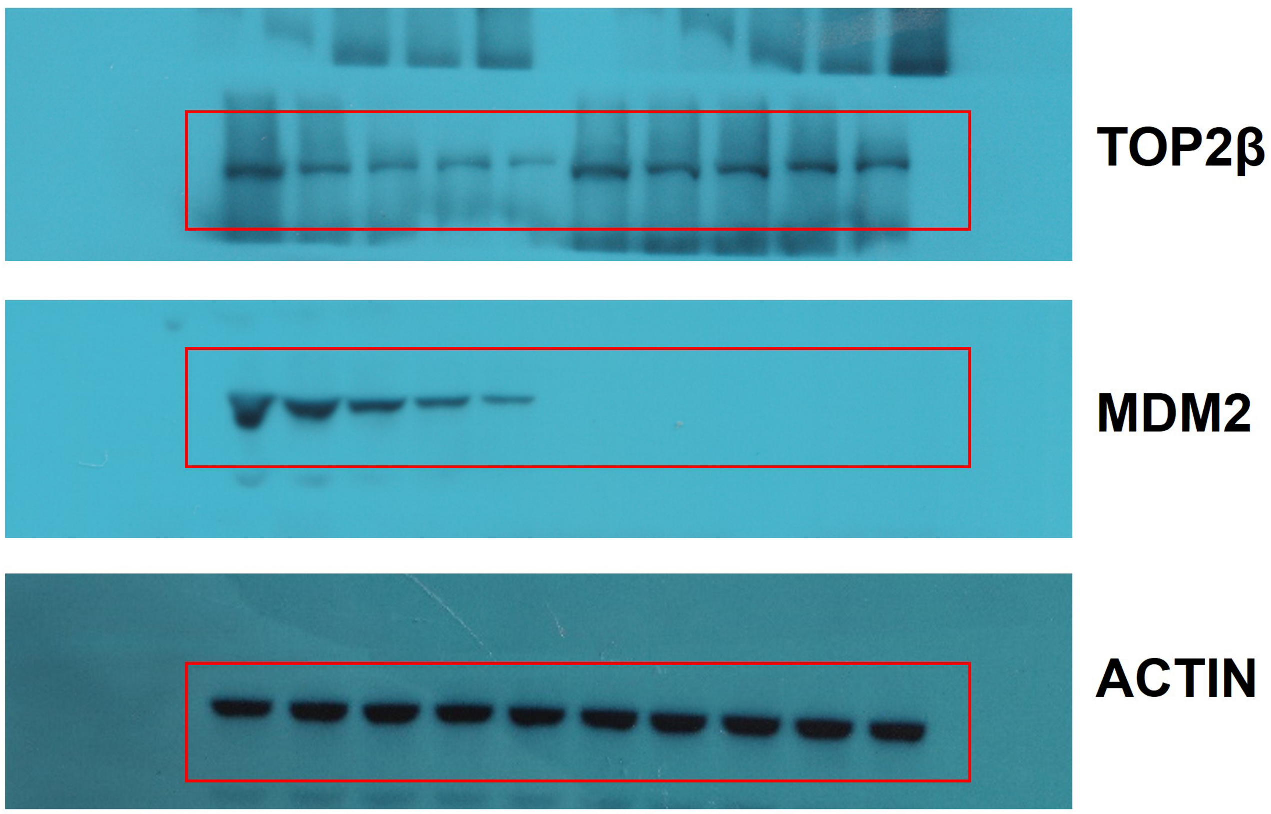

Figure 5C

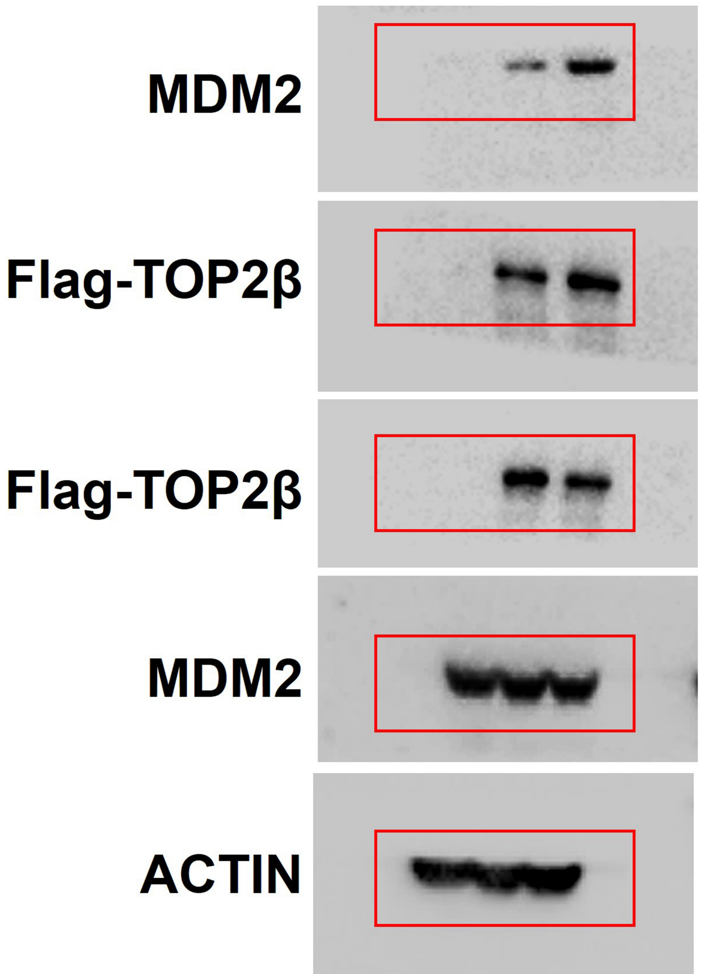

Figure 5D

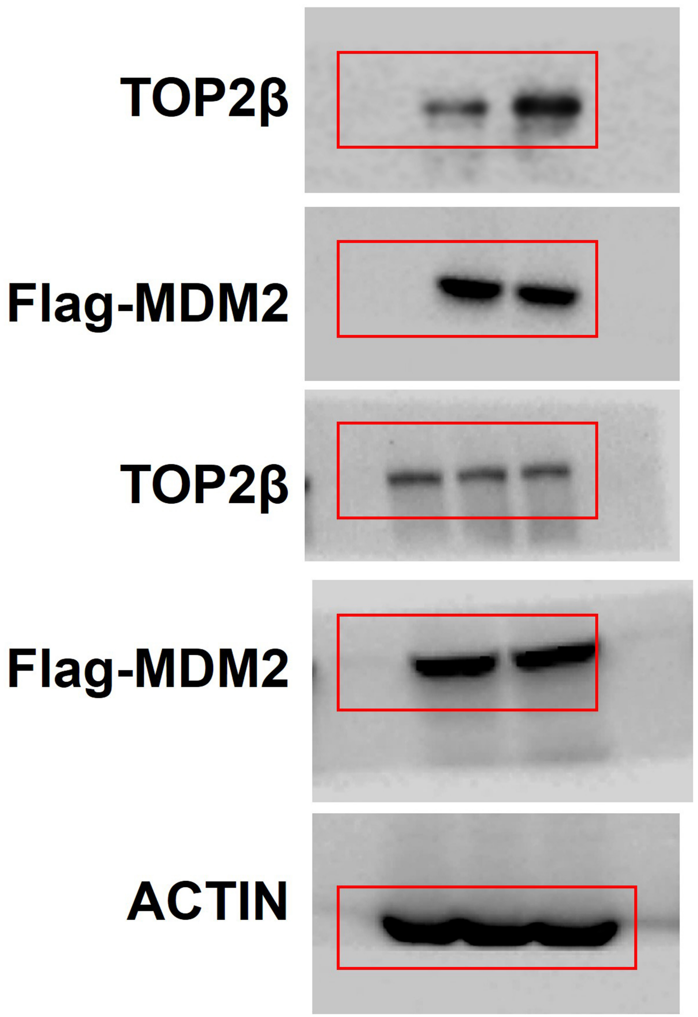

Figure 7I

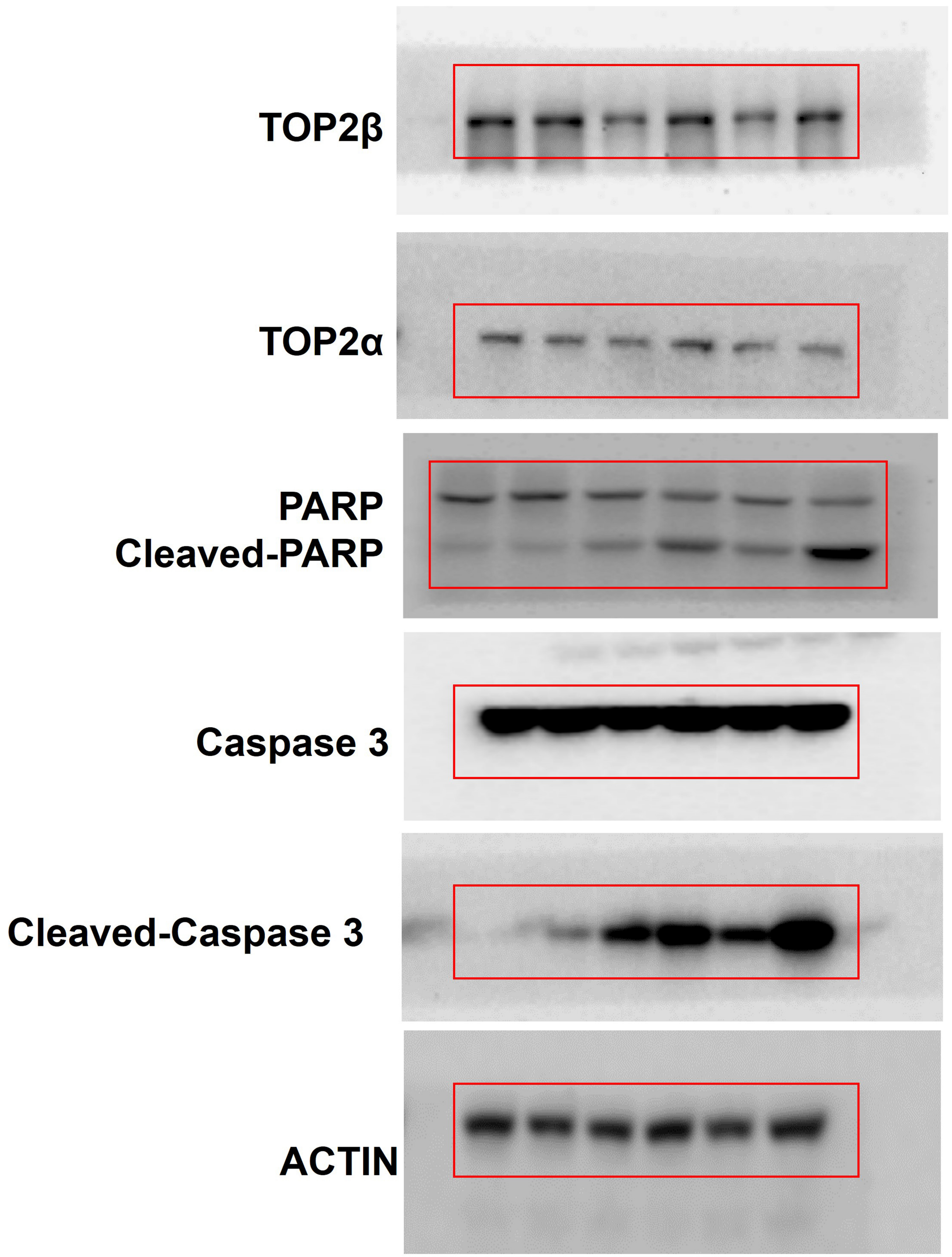

Figure 7J

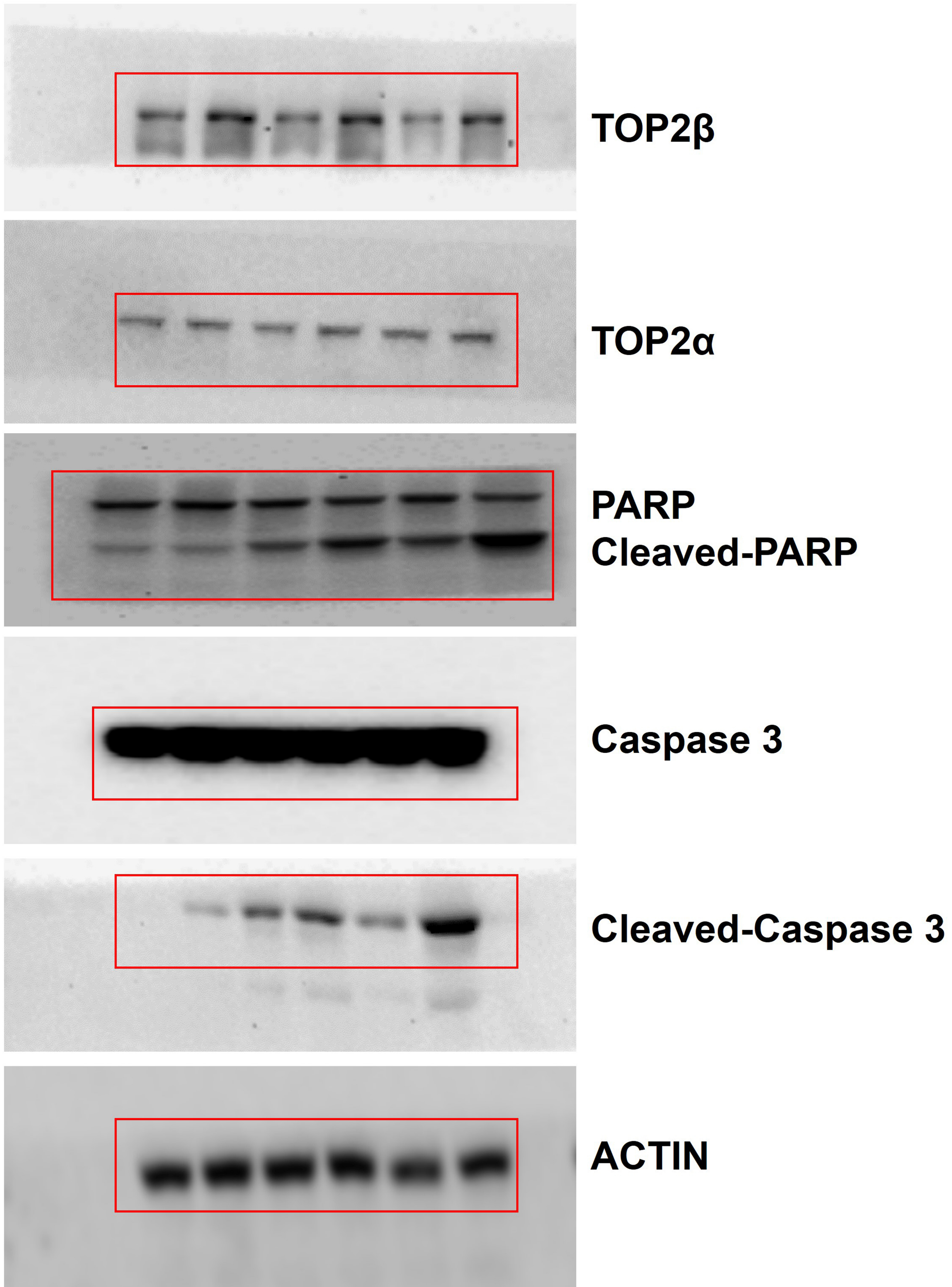

Supplement: Supplementary file 1 — Supplementary Figures [file 41419_2024_6634_MOESM1_ESM.pdf]
